# Global burden and trend of ischemic heart disease and its attributable risk factors among women of childbearing age from 1990 to 2021

**DOI:** 10.3389/fcvm.2026.1695600

**Published:** 2026-03-18

**Authors:** Jieying Liu, Xiaoyan Wang, Xiaofeng He

**Affiliations:** 1 Office of University-Local Government Cooperation, Department of Development Planning, Changzhi Medical College, Changzhi, China; 2 Department of Medical Statistics, School of Public Health, Zhejiang Chinese Medical University, Hangzhou, China; 3 Institute of Evidence-Based Medicine, Heping Hospital Affiliated to Changzhi Medical College, Changzhi, China

**Keywords:** geographical distribution, global burden of disease study, ischemic heart disease, long-term trend, risk factors, women of childbearing age

## Abstract

**Objectives:**

To assess the burden and trends of ischemic heart disease (IHD) and the risk-attributable fractions in women of childbearing age (WCBA) from 1990 to 2021 across 204 countries and territories.

**Methods:**

Data on the number and crude rates of incidence, mortality, disability-adjusted life years (DALYs), and the proportion attributable to risk factors were obtained from the Global Burden of Disease Study 2021. Temporal trends were assessed by calculating the estimated annual percentage change in age-standardized rate.

**Results:**

In 2021, there were 1,349,518 [95% uncertainty interval (UI), 924,620–1,849,024] new cases of IHD among WCBA, resulting in 172,204 (95% UI, 157,564–188,669) deaths and 8,712,835 (95% UI, 7,995,218–9,531,167) DALYs globally. From 1990 to 2021, the age-standardized incidence rate increased by a slight annual change of 0.4%, while the age-standardized mortality and DALY rates declined by 1.04% and 1.02% annually, respectively. Regional analysis revealed the highest IHD burden in North Africa and the Middle East, while Oceania reported the highest mortality-to-incidence ratio. While the age-standardized mortality and DALYs rates of IHD have shown a declining trend in low- and middle-SDI regions, the absolute numbers of deaths and DALYs have risen substantially. Attributable risks, including poor diet, tobacco use, high body mass index, and high blood pressure, were major contributors to IHD-related deaths and DALYs. Notably, the proportion of IHD attributable to high BMI and high blood pressure has increased, particularly in higher-SDI regions.

**Conclusions:**

The burden of IHD among WCBA is rising globally, with significant regional disparities and increasing attributable risks, particularly in low- and middle-SDI regions. These findings highlight the urgent need for targeted, gender-responsive policies and preventive strategies that address modifiable risk factors, strengthen primary healthcare systems, and prioritize cardiovascular health across the life course.

## 1 Introduction

Cardiovascular diseases, particularly ischemic heart disease (IHD), remain the leading cause of death globally and a major contributor to disability (1). Although historically viewed as a condition affecting older men, recent data indicate a troubling rise in IHD incidence and hospitalizations among younger women, especially those of childbearing age (15–49 years), in both high- and low-to-middle-income countries (2–4). For example, Arora et al. reported increasing rates of acute myocardial infarction in young women in the United States, alongside higher in-hospital mortality compared to their male counterparts (5). Additionally, women often present with atypical symptoms—such as fatigue, nausea, and shortness of breath—which may delay diagnosis and treatment, leading to poorer outcomes (6).

Women of childbearing age (WCBA) face distinct cardiovascular risks driven by reproductive and hormonal factors. Conditions such as hypertensive disorders of pregnancy, gestational diabetes, and preeclampsia are now recognized as independent predictors of future cardiovascular disease, including IHD (7, 8). However, these conditions are frequently overlooked in routine cardiovascular risk assessments. Although cardiovascular risk profiles differ across adolescence, early adulthood, and later reproductive years (9), examining WCBA as a unified population allows assessment of the overall burden of IHD occurring during the reproductive life stage and supports alignment with WHO and GBD reporting frameworks. Simultaneously, global increases in obesity, sedentary lifestyles, poor dietary habits, and environmental exposures further heighten IHD risk in this population (10–12). The burden is especially severe in low- and middle-SDI countries, where limited access to quality maternal and preventive healthcare amplifies vulnerability (13).

Despite these alarming trends, comprehensive analyses of IHD burden and its risk factors among WCBA remain scarce (4, 14). Better understanding the magnitude, patterns, and determinants of IHD in this population is essential not only to reduce premature mortality but also to help achieve Sustainable Development Goal (SDG) 3.4, which targets a one-third reduction in noncommunicable disease deaths by 2030 (15). Therefore, this study aims to assess the global, regional, and national burden and trends of IHD and its attributable risks among WCBA from 1990 to 2021, using data from the Global Burden of Disease (GBD) 2021 study (11, 16).

While previous studies have examined IHD in women, our study offers several important and novel contributions (2, 3). First, we focus specifically on WCBA, a group that has been largely underrepresented in global cardiovascular research despite their increasing burden of IHD. Prior studies have typically examined broader female populations without accounting for the unique biological and societal factors affecting women during their reproductive years. Second, we provide the most recent and comprehensive estimates of IHD burden across 204 countries and territories over a 30-year period, offering high-resolution insights into temporal trends and geographic disparities (16). Third, our analysis quantifies the IHD burden attributable to modifiable environmental, behavioral, and metabolic risk factors—an aspect rarely covered in previous work (11). This approach enables more precise identification of region-specific prevention priorities and informs the development of targeted, evidence-based strategies to improve cardiovascular health among women in this critical life stage. Our findings seek to inform policy and guide targeted preventive strategies for improving cardiovascular health among women in this crucial life stage.

## 2 Methods

### 2.1 Overview

The GBD study, led by the Institute for Health Metrics and Evaluation (IHME) with the collaboration of over 10,000 contributors worldwide, aims to assess the comparative impact of diseases, injuries, and risk factors on health loss across different age groups, genders, and geographic regions at specific time points (17). Initiated in 1990, the GBD has been conducted annually using a standardized methodology to produce consistent estimates of disease burden. The GBD 2021 provides detailed, age- and sex-specific data on the incidence, prevalence, mortality, years of life lost (YLLs), years lived with disability (YLDs), and disability-adjusted life years (DALYs) for 371 diseases and injuries, as well as 88 risk factors, covering 204 countries and territories (16).

### 2.2 Data source and data collection

In the GBD 2021, ischemic heart disease (IHD) was estimated using a comprehensive array of data sources, including vital registration systems, verbal autopsy records, population-based health surveys, hospital records, and peer-reviewed publications (16). A detailed description of the input data and methodological approach used for modeling IHD is provided in the Supplementary Materials and methods section, as well as in the official GBD 2021 methods appendices provided by the Institute for Health Metrics and Evaluation (https://www.healthdata.org/gbd/methods-appendices-2021/ischaemic-heart-disease). The international classification of disease (ICD) codes for IHD in GBD 2021 were I20-I21.6 and I21.9-I25.9 based on ICD-10, as well as 410–414.9 and V17.3 under ICD-9 (16). According to the World Health Organization (WHO), women of childbearing age (WCBA) are defined as those aged 15–49 years [https://www.who.int/data/gho/indicator-metadata-registry/imr-details/women-of-reproductive-age-(15-49-years)-population-(thousands)]. For this study, we extracted data on the incidence, deaths, and DALYs for IHD among females aged 15-49 years across 204 countries and territories from 1990 to 2021 from the GBD 2021 using the Global Health Data Exchange (GHDx) query tool (http://ghdx.healthdata.org/gbd-results-tool). All estimates presented in this study, including incidence, mortality, DALYs, and risk-attributable fractions, are accompanied by 95% uncertainty intervals (UIs). These UIs were computed by the GBD study using the 2.5th and 97.5th percentiles of 1,000 draws from the posterior distribution of each modeled estimate, reflecting uncertainty due to sampling error, data limitations, and modeling assumptions (16). As per the GBD 2021 framework, 204 countries and territories were classified into 21 regions based on geographic proximity and epidemiological similarities (Supplementary Table S1) (16).

### 2.3 Socio-demographic index (SDI)

The SDI is a composite measure that reflects a country's development status and shows a strong correlation with health outcomes (16). It is calculated as the geometric mean of three 0-1 indices: fertility rates among females under 25 years, average years of education for individuals aged 15 and above, and lag-distributed income per capita. In 2021, SDI estimates for 204 countries were used to classify nations into five quintiles: low, low-middle, middle, high-middle, and high (Supplementary Table S2).

### 2.4 Risk factors

The GBD 2021 identified 11 risk factors associated with IHD from the 88 risk factors assessed, grouped into three clusters (11): (1) Environmental/Occupational Risks: Air pollution, non-optimal temperature, and other environmental risks; (2) Behavioral Risks: Dietary risks, tobacco use, and low physical activity; (3) Metabolic Risks: High body mass index (BMI), high systolic blood pressure, high fasting plasma glucose, high LDL cholesterol, and kidney dysfunction. The risk-attributable fractions of IHD deaths and DALYs among WCBA were estimated using a comparative risk assessment framework (11). A detailed description of the estimation of IHD burden attributable to risk factor is provided in the Supplementary Materials and methods section.

### 2.5 Statistical analysis

We calculated age-standardized rates (ASRs) of incidence, mortality, and DALYs per 100,000 individuals for IHD among WCBA, using the following formula:

$$\mathrm{ASR}=\frac{\sum_{i=1}^{N}\;a_{i}w_{i}}{\sum_{i=1}^{N}\;w_{i}}\;\times 100,000$$

In this formula, $a_{i}$ represents the age-specific rate for the $i^{th}$ age subgroup, and $w_{i}$ denotes the population count for the same age subgroup *i* based on the GBD 2021 standard population (18). *N* is the total number of age groups. The summation (∑) accounts for all age groups included in the analysis. This method involves weighting the age-specific rates for each population by the proportion of that age group in the standard population, and summing the results to produce a summary rate that is comparable across populations and over time. The 95% confidence intervals (CIs) for these ASRs were computed using the “ageadjust.direct” function from the “epitools” package in R, ensuring consistency with standard epidemiological practice (19). The mortality-to-incidence ratio (MIR) was estimated by dividing the ASMR by the ASIR and multiplying the result by 100.

Estimated annual percentage change (EAPC) was calculated to summarize the temporal change of ASRs over the period 1990–2021 (20). Following established methods, we fitted a log-linear regression model: ln(ASR)=α+β(calendaryear)+ε. Where β represents the annual rate of change on the logarithmic scale. EAPC was then computed as: 100×(exp(β)−1). A positive EAPC with a CI entirely above 0 indicates an increasing trend, whereas a negative EAPC with a CI entirely below 0 indicates a decreasing trend. EAPC provides a summary measure of the average annual change across the full study period and has been widely used in GBD-based burden trend analyses to ensure comparability across regions and countries.

Additionally, a Locally Weighted Scatterplot Smoothing (LOWESS) model was used to examine the correlation between the burden of IHD among WCBA and the SDI across 21 regions (21). LOWESS is a non-parametric smoothing technique that fits locally weighted regression curves without assuming a predefined functional form, and was therefore applied to visually assess potential nonlinear relationships between SDI and IHD burden. Spearman correlation analysis was conducted to calculate the *ρ* indices and *p* values, assessing the relationship between IHD burden and SDI. Spearman's rank correlation was chosen because it does not require normality and is appropriate for evaluating monotonic associations in ecological, region-level data.

All statistics Data analysis was carried out using R software, version 4.1.0 (R Foundation for Statistical Computing). A two-tail *P* < 0.05 was considered statistically significant.

## 3 Results

### 3.1 Global level

In 2021, there were 1,349,518 [95% uncertainty interval (UI), 924,620–1,849,024] new cases of IHD among WCBA worldwide, corresponding to an age-standardized incidence rate (ASIR) of 58.42 (95% UI, 58.30–58.53) per 100,000 population (Table 1). In 2021, IHD was responsible for 172,204 (95% UI, 157,564–188,669) deaths and resulted in 8,712,835 (95% UI, 7,995,218–9,531,167) DALYs for WCBA, with age-standardized mortality rate (ASMR) and age-standardized DALYs rate (ASDR) of 7.59 (95% UI,7.55–7.63) and 388.98 (95% UI, 388.68–389.27), respectively (Table 1). From 1990 to 2021, the absolute numbers of incident cases, deaths, and DALYs due to IHD among WCBA increased by 99.0%, 31.6%, and 28.8%, respectively. In the same period, the global ASIR for IHD increased at an EAPC of 0.4% [95% confidence interval (CI), 0.37–0.43; Table 1]. In contrast, both the ASMR and ASDR declined by an average of 1.04% per year (95% CI, −1.09 to −0.99) and 1.02% per year (95% CI, −1.07 to −0.97), respectively (Table 1). Additionally, the MIR for IHD decreased by 1.43% annually (95% CI, −1.48 to −1.38), dropping from 19.45% in 1990 to 12.99% in 2021 (Supplementary Table S1).

**Table 1:** Global burden of ischemic heart disease among women of childbearing age in 1990 and 2021, and estimated annual percentage changes from 1990 to 2021.

Characteristics	1990		2021		1990–2021
	Number of cases	Age-standardized rate per 100000 population (95% UI)	Number of cases	Age-standardized rate per 100000 population (95% UI)	Estimated annual percentage changes (95% CI)
Incidence					
**Global**	678172 (472491 to 912141)	52.35 (52.20 to 52.50)	1349518 (924620 to 1849024)	58.42 (58.30 to 58.53)	0.40 (0.37 to 0.43)
Socio-demographic index					
High	85554 (59147 to 115696)	32.26 (32.01 to 32.52)	94178 (65561 to 128666)	28.84 (28.62 to 29.06)	−0.56 (-0.66 to -0.46)
High-middle	140642 (95299 to 184934)	49.83 (49.53 to 50.14)	238764 (162167 to 325579)	57.24 (56.97 to 57.50)	0.45 (0.29 to 0.61)
Middle	212330 (145175 to 285255)	52.21 (51.94 to 52.47)	456653 (307466 to 629186)	59.31 (59.11 to 59.51)	0.45 (0.39 to 0.52)
Low-middle	175919 (123126 to 239235)	71.63 (71.23 to 72.02)	396494 (278040 to 539449)	75.01 (74.73 to 75.28)	0.24 (0.18 to 0.31)
Low	63074 (43593 to 85851)	65.63 (65.02 to 66.24)	162452 (113164 to 221971)	67.32 (66.93 to 67.71)	0.10 (0.07 to 0.12)
GBD regions					
High-income Asia Pacific	5083 (3256 to 7338)	9.54 (9.24 to 9.85)	4083 (2416 to 5998)	8.08 (7.78 to 8.38)	-0.62 (−0.71 to −0.53)
Central Asia	7719 (5236 to 10420)	52.12 (50.77 to 53.49)	15125 (10326 to 20377)	54.48 (53.49 to 55.48)	0.19 (0.15 to 0.23)
East Asia	147376 (99237 to 197791)	46.47 (46.19 to 46.74)	263040 (177152 to 361263)	56.76 (56.51 to 57.01)	0.67 (0.45 to 0.89)
South Asia	186412 (126722 to 255304)	79.31 (78.88 to 79.74)	440794 (305180 to 606193)	84.02 (83.73 to 84.31)	0.31 (0.24 to 0.38)
Southeast Asia	29828 (19882 to 39673)	27.07 (26.72 to 27.43)	57230 (38143 to 77248)	26.56 (26.31 to 26.82)	0.03 (−0.02 to 0.08)
Australasia	2315 (1603 to 3177)	37.53 (35.73 to 39.41)	2625 (1675 to 3766)	27.37 (26.16 to 28.62)	-0.98 (−1.18 to −0.78)
Caribbean	6178 (4258 to 8330)	69.41 (67.40 to 71.48)	10238 (7065 to 14096)	73.04 (71.41 to 74.69)	0.15 (0.11 to 0.19)
Central Europe	17669 (11938 to 23226)	52.18 (51.31 to 53.07)	13973 (9548 to 18428)	42.69 (41.87 to 43.53)	-0.89 (−1.07 to −0.71)
Eastern Europe	39054 (26643 to 51108)	63.76 (63.05 to 64.49)	49016 (33466 to 65461)	76.75 (75.95 to 77.56)	0.64 (0.43 to 0.85)
Western Europe	23192 (17213 to 29757)	20.31 (20.01 to 20.62)	18594 (12345 to 25970)	14.34 (14.1 to 14.59)	-1.07 (−1.19 to −0.95)
Andean Latin America	3431 (2305 to 4603)	40.39 (38.81 to 42.03)	8544 (5711 to 11667)	43.89 (42.81 to 44.99)	0.31 (0.27 to 0.35)
Central Latin America	20448 (13860 to 27562)	55.71 (54.81 to 56.62)	42546 (28784 to 58239)	54.00 (53.40 to 54.60)	-0.13 (−0.16 to −0.11)
Southern Latin America	2761 (1815 to 3863)	20.83 (19.93 to 21.75)	4400 (3038 to 6059)	20.84 (20.13 to 21.57)	-0.11 (−0.18 to −0.04)
Tropical Latin America	12155 (8055 to 16364)	32.24 (31.57 to 32.92)	19832 (13570 to 26432)	27.61 (27.17 to 28.06)	-0.24 (−0.33 to −0.15)
North Africa and Middle East	82985 (57935 to 112435)	125.3 (124.29 to 126.32)	228679 (157914 to 312013)	126.35 (125.74 to 126.97)	0.01 (−0.03 to 0.04)
High-income North America	40210 (25739 to 57848)	46.18 (45.65 to 46.72)	27949 (20362 to 37203)	26.73 (26.37 to 27.10)	-2.39 (−2.56 to −2.23)
Oceania	619 (424 to 831)	44.89 (40.80 to 49.30)	1643 (1108 to 2208)	46.03 (43.46 to 48.72)	0.13 (0.09 to 0.16)
Central Sub-Saharan Africa	5920 (4096 to 8093)	56.84 (55.15 to 58.58)	16098 (11077 to 22351)	55.77 (54.75 to 56.81)	-0.13 (−0.17 to −0.10)
Eastern Sub-Saharan Africa	17773 (11987 to 24213)	50.89 (50.01 to 51.79)	48213 (32456 to 65619)	52.61 (52.05 to 53.18)	0.10 (0.09 to 0.11)
Southern Sub-Saharan Africa	8302 (5679 to 11288)	72.86 (71.01 to 74.75)	15920 (10711 to 21969)	68.29 (67.05 to 69.55)	-0.35 (−0.42 to −0.27)
Western Sub-Saharan Africa	18742 (12671 to 25586)	52.78 (51.88 to 53.68)	60976 (41131 to 83312)	58.99 (58.43 to 59.55)	0.4 (0.38 to 0.42)
Death					
**Global**	130835 (117793 to 144303)	10.18 (10.12 to 10.24)	172204 (157564 to 188669)	7.59 (7.55 to 7.63)	-1.04 (−1.09 to −0.99)
Socio-demographic index					
High	10961 (10660 to 11332)	4.23 (4.13 to 4.32)	8492 (7795 to 9416)	2.54 (2.48 to 2.6)	-1.68 (−1.75 to −1.61)
High-middle	21321 (18973 to 23844)	7.58 (7.46 to 7.70)	17469 (15511 to 19751)	4.09 (4.02 to 4.17)	-2.42 (−2.78 to −2.06)
Middle	43214 (39723 to 47177)	10.65 (10.54 to 10.77)	58407 (53017 to 63798)	7.76 (7.69 to 7.83)	-1.07 (−1.14 to −1.00)
Low-middle	43587 (36498 to 50261)	17.56 (17.37 to 17.75)	66530 (58309 to 75765)	12.64 (12.53 to 12.75)	-0.99 (−1.06 to −0.91)
Low	11613 (9246 to 14562)	12.12 (11.87 to 12.38)	21162 (17503 to 24825)	8.77 (8.63 to 8.91)	-1.25 (−1.37 to −1.13)
GBD regions					
High-income Asia Pacific	1039 (967 to 1120)	1.97 (1.83 to 2.11)	479 (455 to 505)	0.83 (0.74 to 0.93)	-2.98 (−3.13 to −2.82)
Central Asia	1962 (1845 to 2090)	13.35 (12.66 to 14.06)	2263 (1913 to 2650)	8.37 (7.98 to 8.77)	-2.46 (−2.94 to −1.97)
East Asia	23284 (18540 to 28723)	7.27 (7.16 to 7.38)	17416 (13333 to 22526)	3.75 (3.68 to 3.81)	-2.08 (−2.24 to −1.93)
South Asia	42027 (33824 to 49269)	17.80 (17.60 to 18.00)	72041 (61515 to 82500)	13.88 (13.76 to 14.00)	-0.77 (−0.90 to −0.64)
Southeast Asia	15756 (13381 to 18482)	14.40 (14.14 to 14.66)	22563 (18912 to 27176)	10.54 (10.38 to 10.70)	-0.97 (−1.01 to −0.93)
Australasia	199 (187 to 212)	3.33 (2.8 to 3.92)	116 (107 to 127)	1.19 (0.95 to 1.48)	-3.26 (−3.41 to −3.11)
Caribbean	1143 (1010 to 1305)	13.14 (12.27 to 14.07)	1313 (1034 to 1701)	9.38 (8.80 to 9.98)	-0.91 (−1.15 to −0.67)
Central Europe	2951 (2864 to 3041)	8.09 (7.75 to 8.45)	1055 (936 to 1169)	2.77 (2.58 to 2.98)	-3.89 (−4.08 to −3.71)
Eastern Europe	4661 (4419 to 4810)	7.49 (7.23 to 7.75)	4246 (3684 to 4936)	5.87 (5.66 to 6.09)	-1.93 (−2.75 to −1.1)
Western Europe	3536 (3437 to 3641)	3.17 (3.05 to 3.3)	1307 (1258 to 1353)	0.98 (0.92 to 1.04)	-3.77 (−3.89 to −3.64)
Andean Latin America	678 (578 to 810)	7.93 (7.26 to 8.66)	677 (519 to 868)	3.61 (3.30 to 3.93)	-2.82 (−3.23 to −2.42)
Central Latin America	2724 (2648 to 2798)	7.57 (7.24 to 7.91)	4324 (3659 to 4948)	5.54 (5.35 to 5.74)	-1.14 (−1.54 to −0.74)
Southern Latin America	737 (690 to 785)	5.56 (5.11 to 6.05)	451 (416 to 489)	2.13 (1.91 to 2.38)	-2.81 (−3.17 to −2.44)
Tropical Latin America	3510 (3404 to 3622)	9.61 (9.24 to 10.00)	4031 (3809 to 4238)	5.37 (5.18 to 5.57)	-2.1 (−2.2 to −2.01)
North Africa and Middle East	16338 (13748 to 19397)	24.25 (23.83 to 24.69)	23781 (19111 to 29544)	13.69 (13.49 to 13.89)	-1.92 (−1.97 to −1.86)
High-income North America	4130 (4036 to 4223)	4.95 (4.78 to 5.13)	3556 (3419 to 3722)	3.29 (3.16 to 3.42)	-1.27 (−1.45 to −1.10)
Oceania	246 (162 to 358)	18.16 (15.55 to 21.11)	592 (416 to 808)	16.62 (15.08 to 18.28)	-0.31 (−0.45 to −0.17)
Central Sub-Saharan Africa	595 (364 to 882)	6.05 (5.50 to 6.64)	1317 (831 to 1936)	4.79 (4.50 to 5.11)	-0.88 (−0.99 to −0.78)
Eastern Sub-Saharan Africa	2242 (1759 to 3058)	5.93 (5.64 to 6.23)	4388 (3582 to 5427)	4.47 (4.31 to 4.63)	-1.2 (−1.31 to −1.09)
Southern Sub-Saharan Africa	867 (763 to 982)	7.51 (6.94 to 8.12)	1197 (1006 to 1395)	5.23 (4.89 to 5.59)	-0.31 (−1.02 to 0.40)
Western Sub-Saharan Africa	2210 (1697 to 2859)	6.81 (6.49 to 7.15)	5092 (3858 to 6670)	5.25 (5.09 to 5.42)	-0.89 (−1.05 to −0.73)
DALYs					
**Global**	6762284 (6070763 to 7486370)	518.53 (518.08 to 518.98)	8712835 (7995218 to 9531167)	388.98 (388.68 to 389.27)	-1.02 (−1.07 to −0.97)
Socio-demographic index					
High	544726 (527271 to 563780)	209.42 (208.77 to 210.07)	421677 (386919 to 466986)	128.29 (127.84 to 128.75)	-1.61 (−1.67 to −1.55)
High-middle	1089471 (976357 to 1217090)	382.38 (381.55 to 383.22)	870921 (773955 to 989662)	209.78 (209.27 to 210.3)	-2.34 (−2.67 to −2.00)
Middle	2254490 (2066077 to 2458937)	543.56 (542.73 to 544.38)	2937394 (2670794 to 3219524)	397.45 (396.93 to 397.97)	-1.05 (−1.11 to −1.00)
Low-middle	2269362 (1886858 to 2628466)	895.60 (894.26 to 896.94)	3382078 (2969695 to 3847527)	639.98 (639.19 to 640.77)	-1.03 (−1.10 to −0.96)
Low	597283 (476263 to 754396)	604.75 (602.97 to 606.54)	1093475 (906672 to 1280899)	441.08 (440.12 to 442.05)	-1.21 (−1.34 to −1.09)
GBD regions					
High-income Asia Pacific	54306 (50214 to 58408)	104.35 (103.35 to 105.36)	24699 (23394 to 26293)	44.63 (43.97 to 45.31)	-2.96 (−3.10 to −2.81)
Central Asia	102554 (96202 to 109174)	674.49 (669.70 to 679.31)	114610 (96962 to 133957)	425.62 (422.84 to 428.41)	-2.42 (−2.89 to −1.94)
East Asia	1214345 (969703 to 1497010)	372.64 (371.86 to 373.42)	879201 (681405 to 1131619)	196.31 (195.82 to 196.79)	-2.02 (−2.17 to −1.87)
South Asia	2180662 (1745901 to 2556354)	906.58 (905.19 to 907.96)	3650598 (3122187 to 4182589)	701.33 (700.51 to 702.16)	-0.81 (−0.93 to −0.70)
Southeast Asia	829480 (699521 to 978064)	741.33 (739.50 to 743.16)	1136974 (957056 to 1367650)	539.16 (538.03 to 540.3)	-1.00 (−1.05 to −0.95)
Australasia	9765 (9200 to 10386)	161.75 (157.99 to 165.59)	5725 (5299 to 6243)	58.40 (56.63 to 60.21)	-3.27 (−3.43 to −3.12)
Caribbean	57507 (50730 to 65423)	649.70 (643.53 to 655.93)	65627 (51876 to 84857)	472.88 (468.74 to 477.05)	-0.81 (−1.05 to −0.57)
Central Europe	144655 (140081 to 149234)	394.82 (392.42 to 397.24)	52189 (46671 to 57770)	139.45 (138.04 to 140.87)	-3.79 (−3.97 to −3.61)
Eastern Europe	227141 (215453 to 234581)	359.14 (357.39 to 360.90)	204922 (177635 to 237929)	286.49 (285.02 to 287.98)	-1.87 (−2.69 to −1.05)
Western Europe	175541 (170126 to 180906)	157.72 (156.87 to 158.58)	65773 (62914 to 68708)	50.11 (49.67 to 50.56)	-3.69 (−3.8 to −3.57)
Andean Latin America	36717 (31461 to 43737)	418.79 (413.9 to 423.72)	36024 (28048 to 45833)	193.19 (190.95 to 195.46)	-2.76 (−3.16 to −2.37)
Central Latin America	141936 (138140 to 145836)	383.09 (380.75 to 385.43)	219963 (187805 to 249696)	284.38 (283.02 to 285.75)	-1.06 (−1.47 to −0.66)
Southern Latin America	37266 (35039 to 39631)	281.30 (278.02 to 284.61)	22573 (20942 to 24480)	107.83 (106.21 to 109.48)	-2.83 (−3.22 to −2.44)
Tropical Latin America	173830 (168546 to 179487)	465.93 (463.34 to 468.53)	198422 (188065 to 207894)	265.85 (264.48 to 267.22)	-2.03 (−2.13 to −1.93)
North Africa and Middle East	857579 (719009 to 1016565)	1235.00 (1231.99 to 1238.02)	1210045 (972971 to 1496997)	698.94 (697.51 to 700.36)	-1.9 (−1.95 to −1.85)
High-income North America	201320 (196502 to 206614)	238.09 (236.87 to 239.32)	173095 (166276 to 181716)	160.87 (159.98 to 161.76)	-1.25 (−1.42 to −1.08)
Oceania	12255 (7975 to 17977)	881.23 (862.71 to 900.06)	29482 (20808 to 40463)	819.68 (808.76 to 830.72)	-0.26 (−0.40 to −0.11)
Central Sub-Saharan Africa	29852 (18665 to 43767)	292.69 (288.84 to 296.57)	66431 (42413 to 95559)	234.25 (232.17 to 236.34)	-0.84 (−0.95 to −0.74)
Eastern Sub-Saharan Africa	120137 (94623 to 162409)	306.06 (303.99 to 308.13)	237261 (193332 to 292520)	234.38 (233.26 to 235.51)	-1.14 (−1.25 to −1.03)
Southern Sub-Saharan Africa	45076 (39720 to 51070)	382.67 (378.56 to 386.81)	59862 (50411 to 69789)	258.91 (256.50 to 261.34)	-0.49 (−1.26 to 0.28)
Western Sub-Saharan Africa	110358 (84720 to 142528)	326.78 (324.54 to 329.03)	259358 (197639 to 339691)	258.23 (257.08 to 259.39)	-0.8 (−0.96 to −0.64)

### 3.2 Regional level

**Figure 1:**
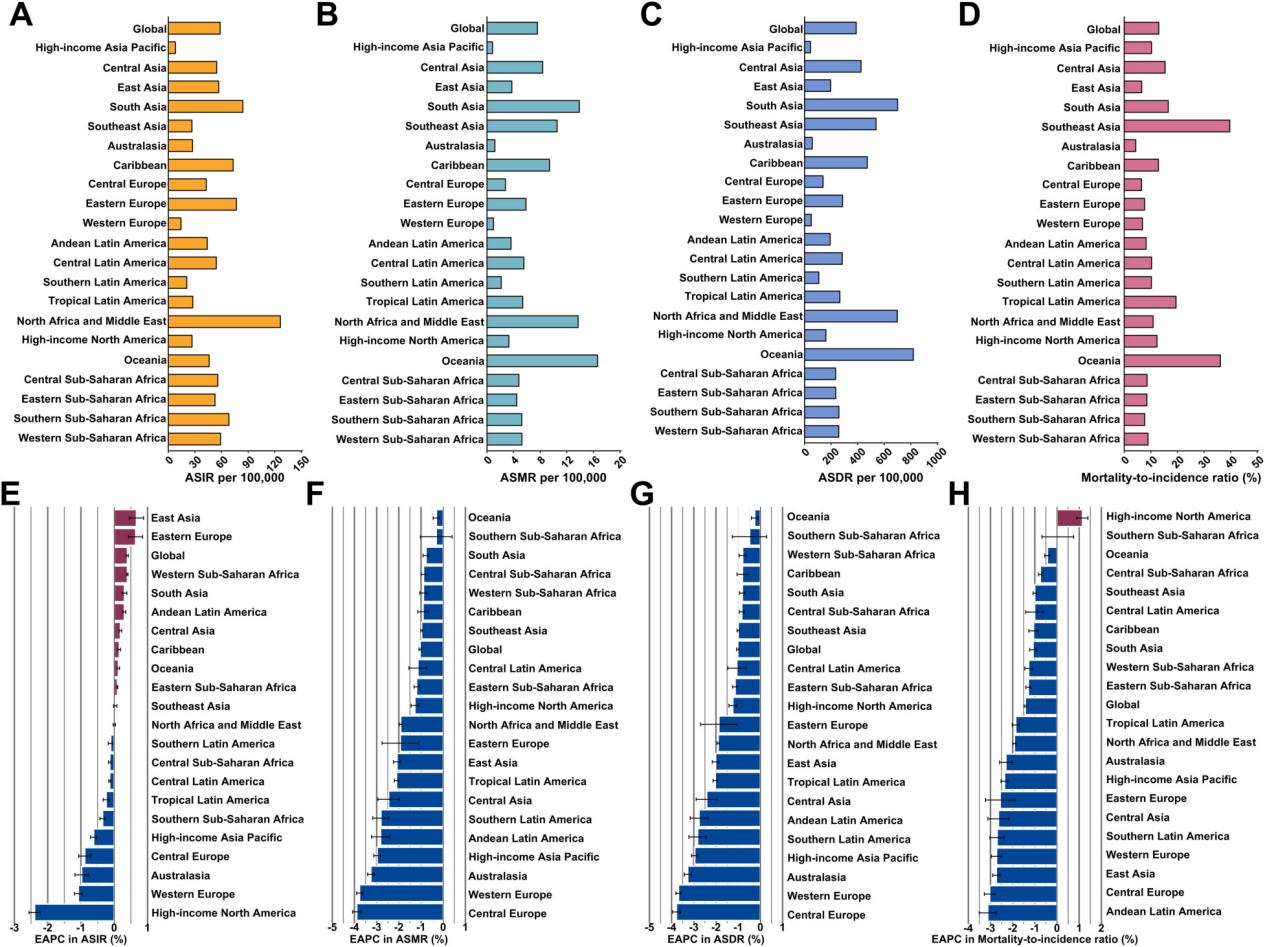
The burden and trend of ischemic heart disease among women of childbearing age in 2021, along with their estimated annual percentage changes from 1990 to 2021, globally and across 21 GBD regions. **(A)** Age-standardized incidence rate. **(B)** Age-standardized mortality rate. **(C)** Age-standardized DALYs rate. **(D)** mortality-to-incidence ratio. **(E)** EAPC in age-standardized incidence rate. **(F)** EAPC in age-standardized mortality rate. **(G)** EAPC in age-standardized DALYs rate. **(H)** EAPC in mortality-to-incidence ratio. DALY = Disability-adjusted life-years; EAPC = estimated annual percentage change.

In 2021, among the 21 GBD regions, North Africa and the Middle East had the highest ASIR of 126.35 per 100,000 population (95% UI, 125.74–126.97; Figure 1A). In contrast, Oceania had both the highest ASMR of 16.62 (95% UI, 15.08–18.28) and the highest ASDR of 819.68 (95% UI, 808.76–830.72) (Figures 1B,C). Southeast Asia reported the highest MIR at 39.68% (Figure 1D, Supplementary Table S1). Between 1990 and 2021, East Asia experienced the fastest annual increase in ASIR, at 0.67% (95% CI, 0.45–0.89), while High-income North America showed the largest decrease, with an EAPC of −2.39% (95% CI, −2.56 to −2.23) (Figure 1E and Table 1). The ASMR and ASDR for IHD decreased across 21 GBD regions, with the most significant reductions observed in Central Europe and the smallest in Oceania (Figures 1F,G). Regarding the MIR, it declined in nearly all GBD regions, with the largest decrease in Andean Latin America [EAPC = −3.12% (95% CI, −3.50 to −2.74)]. Only High-income North America saw an increase in MIR, rising by 1.15% annually (95% CI, 0.90–1.39%), while it remained stable in Southern Sub-Saharan Africa [EAPC = 0.04% (95% CI, −0.67 to 0.75); Figure 1H].

### 3.3 National and territorial level

**Figure 2:**
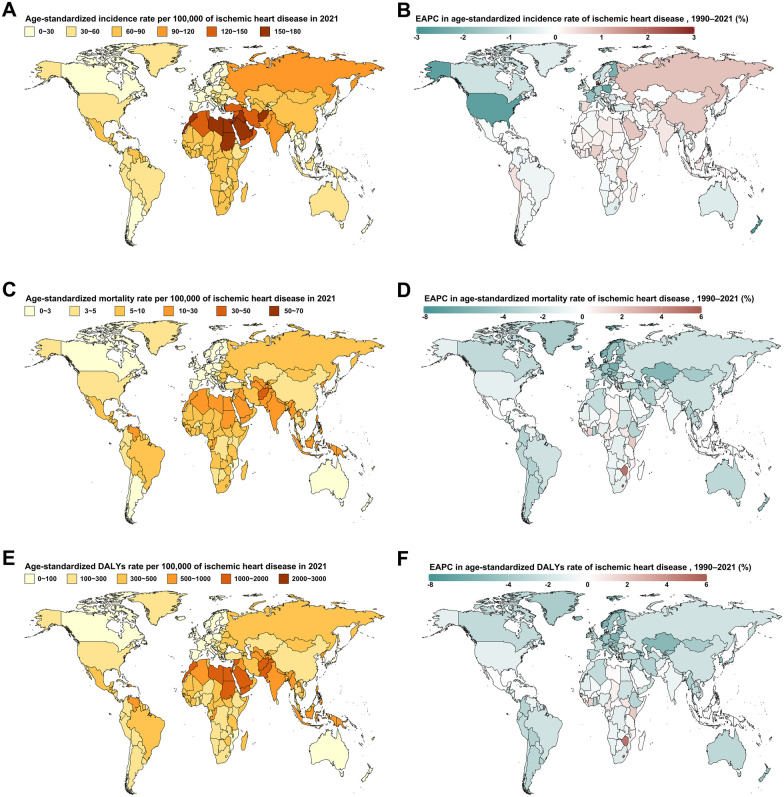
Age-standardized rates for the burden of ischemic heart disease among women of childbearing age in 2021, and their estimated annual percentage changes from 1990 to 2021, by country and territory. Age-standardized incidence rate **(A)** and its estimated annual percentage change **(B)** Age-standardized mortality rate **(C)** and its estimated annual percentage change **(D)** Age-standardized DALYs rate **(E)** and its estimated annual percentage change **(F).**

In 2021, the ASIR of IHD among WCBA varied widely, ranged from 6.94 (95% UI, 5.98 to 8.04) cases per 100,000 population in Portugal to 168.32 (95% UI, 165.65–171.02) cases per 100,000 population in Iraq (Figure 2A). From 1990 to 2021, 112 countries and territories saw an increase in ASIR, with Denmark experiencing the fastest rise at an EAPC of 2.14% (95% CI, 1.47–2.82; Figure 2B). The highest ASMR was recorded in Nauru, with 61.13 (95% UI, 3.88–285.51) deaths per 100,000 population, followed by the Marshall Islands at 34.85 (95% UI, 10.75–84.8) deaths per 100,000 population (Figure 2C). Similarly, Nauru and the Marshall Islands had the highest ASDR, with 2973.93 (95% UI, 2318.35–3765.08) and 1683.33 (95% UI, 1471.46–1917.86), respectively (Figure 2D). Between 1990 and 2021, 19 countries and territories experienced an increase in ASMR, with Lesotho and Zimbabwe recording the fastest rises, exceeding 5% per year (Figure 2E). Additionally, 21 countries and territories saw an increase in ASDR, with Lesotho and Zimbabwe again showing the largest increases (Figure 2F).

### 3.4 Association with the SDI

**Figure 3:**
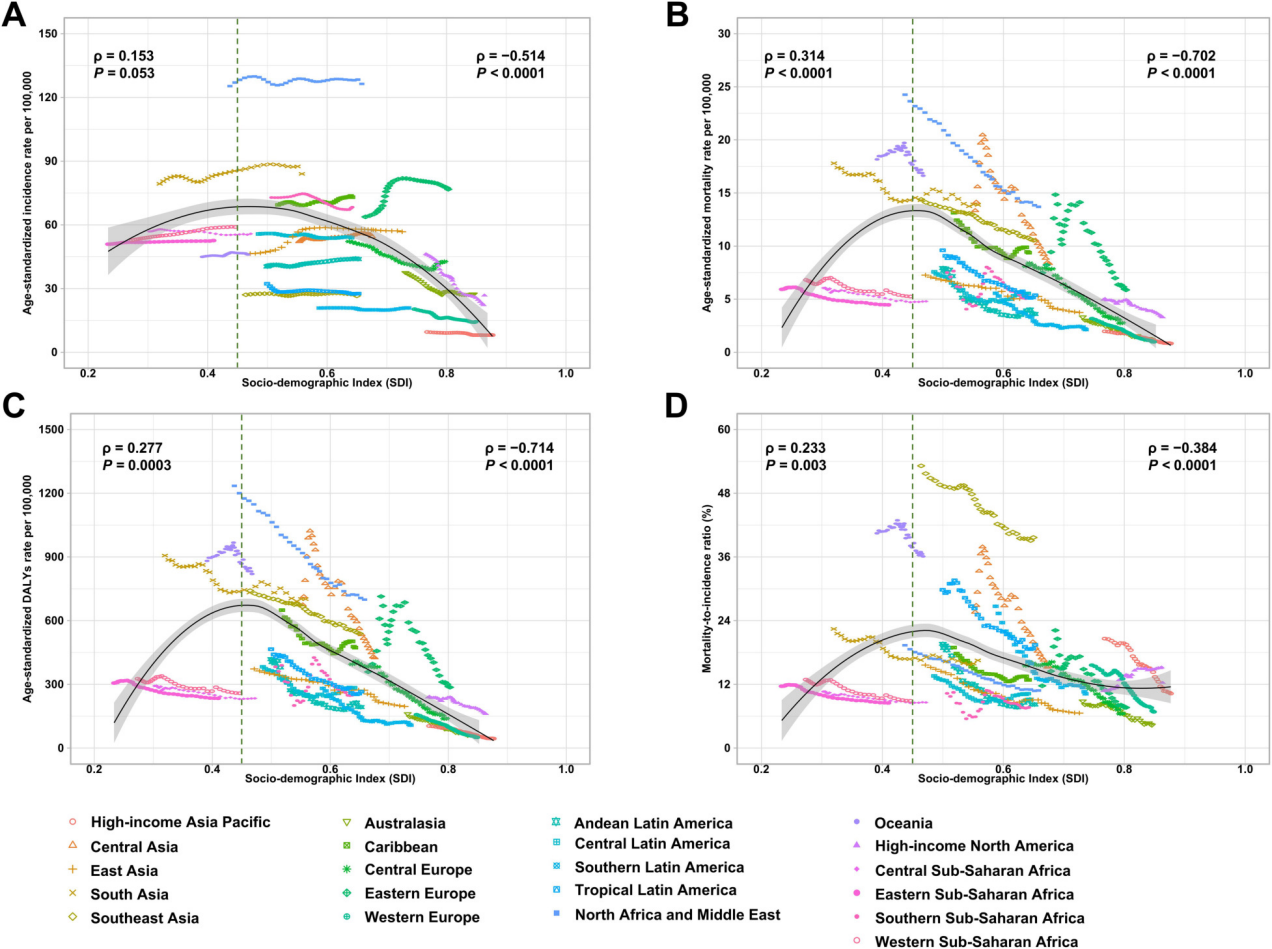
Age-standardized rates of incidence **(A)**, mortality **(B)**, and DALY **(C)**, along with the mortality-to-incidence ratio **(D)** for ischemic heart disease among women of childbearing age, across 21 GBD regions, by socio-demographic index (SDI), from 1990 to 2021. Expected values with 95% confidence intervals (CI), based on SDI and disease rates across all locations, are represented by the solid line and shaded area. Thirty-two data points are plotted for each region, showing the observed disease rate for each year from 1990 to 2021. Points above the solid line indicate a higher-than-expected burden, while points below the line represent a lower-than-expected burden. DALY = Disability-Adjusted Life Years; GBD = Global Burden of Disease Study.

The relationship between the burden of IHD and the SDI exhibited an inverted “U” shape (Figure 3). Specifically, the ASIR, ASMR, ASDR, and MIR initially increased with rising SDI, peaking around an SDI value of 0.45, before declining. Notably, regions such as North Africa and the Middle East, South Asia, and Eastern Europe exhibited higher-than-expected ASIR values (Figure 3A). Similarly, North Africa and the Middle East, Oceania, Central Asia, South Asia, Southeast Asia, and Eastern Europe showed higher-than-expected ASMR and ASDR (Figures 3B,C). Furthermore, Southeast Asia and Oceania had significantly elevated MIR for IHD compared to expected values (Figure 3D).

Although WCBA were analyzed as a single group (15–49 years) for consistency with WHO definitions and GBD reporting, age-specific burden and case-fatality patterns across narrower age categories are provided to reflect within-group heterogeneity. Across all SDI regions, the rates of incidence, mortality, and DALYs increased with age (Supplementary Figure S1A-C). However, the highest MIR for IHD were observed in the 15-19 years age group, except in high-SDI regions, where females aged 45-49 years experienced the highest MIR (Supplementary Figure S1D). Notably, substantial variation was observed across age subgroups within WCBA, with incidence, mortality, and DALY rates increasing with age, while younger women exhibited relatively higher MIR (Supplementary Figure S1), underscoring the heterogeneity of cardiovascular risk within the reproductive age range. From 1990 to 2021, the burden of ischemic heart disease among WCBA showed substantial declines in mortality and DALY rates, with marked heterogeneity by age group and SDI. Across nearly all SDI strata and age groups, mortality and DALY rates decreased, with the largest reductions observed in high and high-middle SDI regions, particularly among younger women. In contrast, low and low-middle SDI regions experienced smaller declines, and improvements were attenuated among women aged 35–49 years (Supplementary Figure S2). Trends in incidence were more variable. While incidence declined or remained stable in most middle to low SDI regions, high and high-middle SDI settings showed stable or modestly increasing incidence, especially among women aged 30–44 years. Overall, these findings indicate continued global progress in reducing fatal and disability-related IHD burden among WCBA, but also reveal persistent socioeconomic disparities and a discordance between incidence and outcome trends in higher SDI regions, suggesting improved survival alongside ongoing or emerging exposure to cardiovascular risk factors (Supplementary Figure S2). Furthermore, between 1990 and 2021, the absolute burden of IHD among WCBA increased, with the most significant rise occurring in low- and middle-SDI regions, particularly in low-SDI areas (Supplementary Figure S3).

### 3.5 Risk-attributable fraction

**Figure 4:**
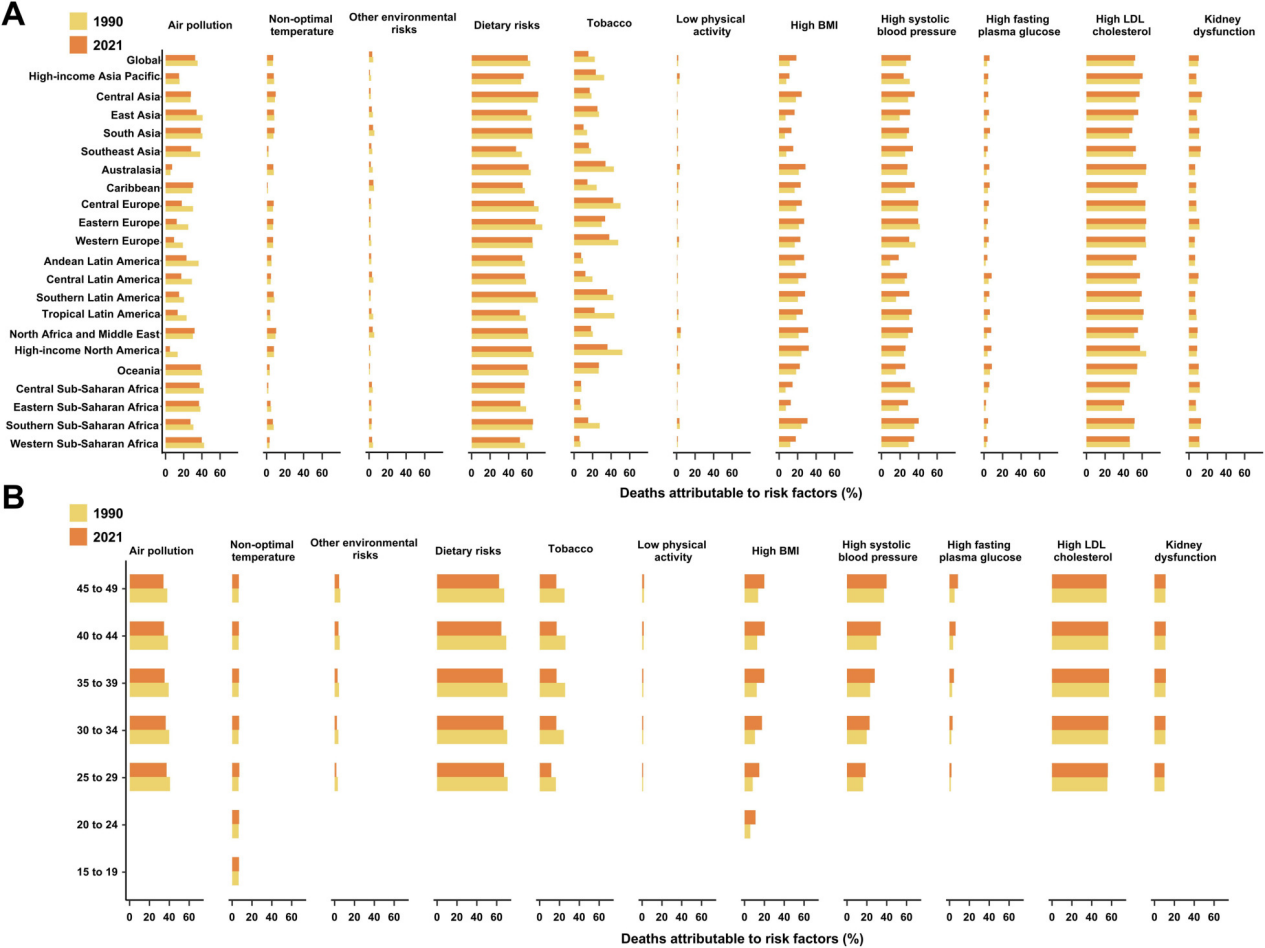
The proportion of deaths attributable to risk factors for ischemic heart disease among women of childbearing age by locations **(A)** and age group globally **(B)** in 2021. In the GBD 2021, certain risk factors were modeled with lower age restrictions of 20 or 30 years, which resulted in the absence of estimates for these risk factors in age groups such as 15-19 years, 20-24 years, and 25-29 years.

Globally, a substantial proportion of deaths and DALYs due to IHD in WCBA were attributable to 11 attributable risks identified in the GBD 2021 (Figure 4A). Among all evaluated risks, dietary risks represented the largest contributor to IHD mortality in 2021, followed by high LDL cholesterol, high systolic blood pressure, air pollution, and high body mass index (BMI). These five factors together accounted for the majority of attributable IHD deaths and DALYs in this population, highlighting the dominant role of metabolic and behavioral risks (Supplementary Figure S4). As for environmental and occupational risks, air pollution remained a major contributor, accounting for 32.5% (95% UI, 25.2 to 39.6) of IHD deaths in 2021—a decline from 35.2% in 1990. Non-optimal temperature contributed 6.8% (95% UI, 4.4 to 9.6), slightly up from 6.4% (95% UI, 4.6 to 8.7), while other environmental risks declined from 4.5% (95% UI, −0.6 to 10.0) to 3.5% (95% UI, −0.5 to 7.7) over the same period. Behavioral risks also played a significant role. Dietary risks were the leading contributor, responsible for 60.4% (95% UI, −12.2 to 85.1) of IHD deaths in 2021, though this marked a decrease from 63.3% (95% UI, −9.3 to 85.3) in 1990. Tobacco use declined considerably, from 22.3% (95% UI, 18.1 to 26.7) in 1990 to 15.4% (95% UI, 12.3 to 18.9) in 2021. Conversely, low physical activity saw a modest increase, contributing 1.5% (95% UI, 0.7 to 2.2) of deaths in 2021, up from 1.3% (95% UI, 0.7 to 1.9) in 1990. Metabolic risks showed a mixed trend. High body mass index (BMI) rose from 11.5% (95% UI, 4.6 to 18.1) in 1990 to 18.6% (95% UI, 7.9 to 28.6) in 2021. Similarly, high systolic blood pressure increased from 26.7% (95% UI, 19.1 to 34.1) to 31.4% (95% UI, 23.4 to 39.2), and high fasting plasma glucose rose from 3.3% (95% UI, 2.8 to 4.0) to 5.9% (95% UI, 5.0 to 7.1). High LDL cholesterol remained a major factor, slightly increasing from 51.1% (95% UI, 39.2 to 61.7) in 1990 to 52.6% (95% UI, 40.6 to 63.4) in 2021. Kidney dysfunction also showed a small rise, from 10.0% (95% UI, 7.1 to 13.1) to 10.5% (95% UI, 7.3 to 14.0).

**Figure 5:**
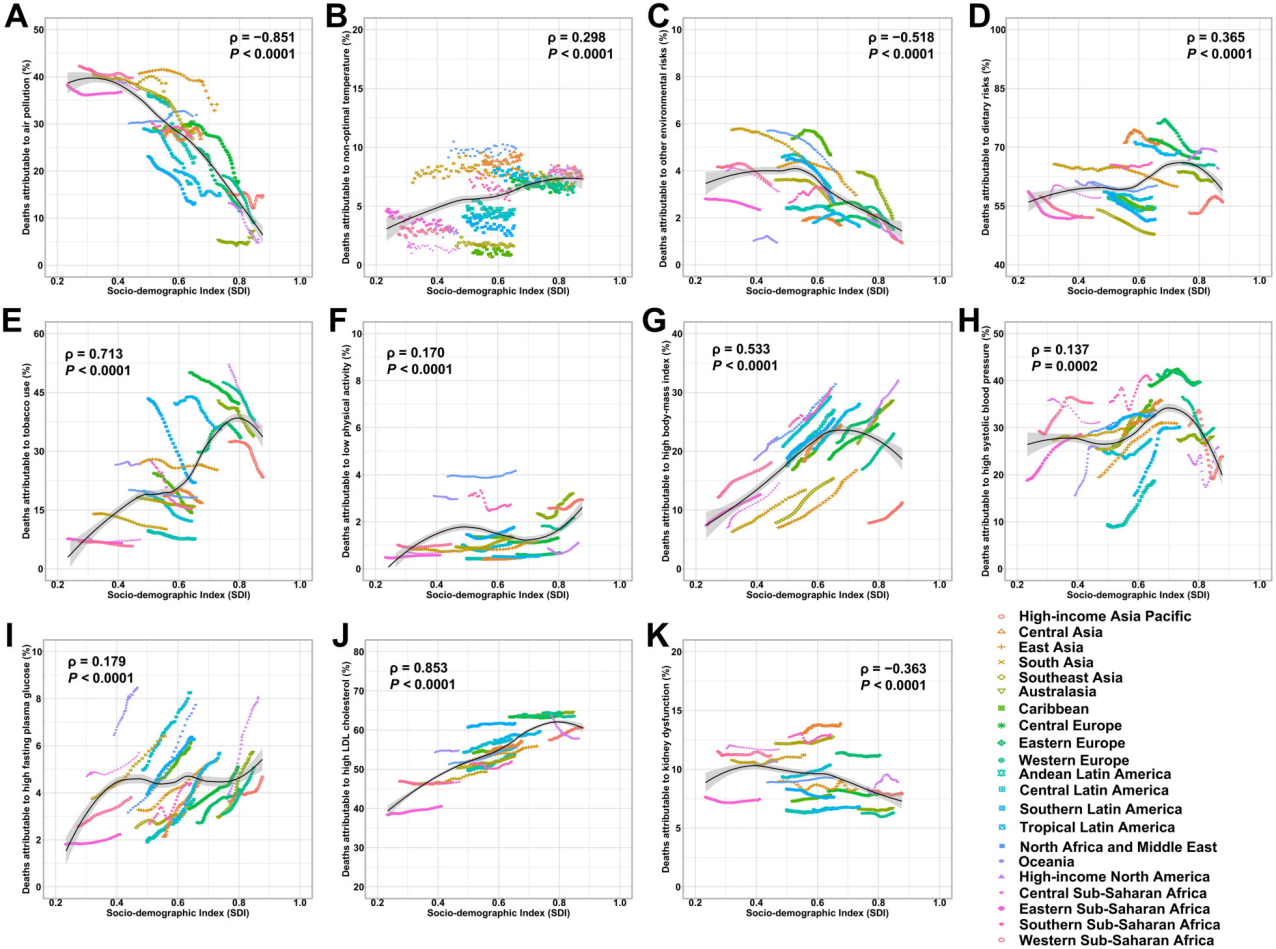
The association between proportion of deaths from ischemic heart disease attributable to risk factors with socio-demographic index (SDI) from 1990 to 2021, by 21 GBD regions. **(A)** Proportion of deaths attributable to air pollution. **(B)** Proportion of deaths attributable to non-optimal temperature. **(C)** Proportion of deaths attributable to other environmental risks. **(D)** Proportion of deaths attributable to dietary risks. **(E)** Proportion of deaths attributable to tobacco use. **(F)** Proportion of deaths attributable to low physical activity. **(G)** Proportion of deaths attributable to high body-mass index. **(H)** Proportion of deaths attributable to high systolic blood pressure. **(I)** Proportion of deaths attributable to high fasting plasma glucose. **(J)** Proportion of deaths attributable to high LDL cholesterol. **(K)** Proportion of deaths attributable to kidney dysfunction. The black line indicates expected values.

The proportion of IHD attributable to air pollution, non-optimal temperature, and dietary risks decreased with age (Figure 4B). In contrast, the contributions of other environmental risks, tobacco use, low physical activity, and metabolic risk factors—including high BMI, high systolic blood pressure, high fasting plasma glucose, and high LDL cholesterol—tended to increase with age. More importantly, a negative association between the risk-attributable proportion of IHD and the SDI was observed only for air pollution and kidney dysfunction. For the remaining nine risk factors, a positive correlation with SDI was found (Figure 5). Furthermore, the patterns of risk-attributable proportions for IHD-related deaths closely mirror those for DALYs attributable to IHD (Supplementary Figure S5).

## 4 Discussion

### 4.1 Principal findings

This study provides an in-depth analysis of the global burden and trends of IHD among WCBA, utilizing data from the GBD 2021. The results highlight both the rising incidence of IHD in this population and the complex interplay of socioeconomic and risk factors that contribute to its burden. In 2021, IHD led to 1,349,518 (95% UI, 924,620 to 1,849,024) new cases and 172,204 (95% UI, 157,564 to 188,669) deaths globally, with a dramatic 99.0% increase in incident cases over the past three decades. Although ASMR and ASDR have declined, the absolute numbers of deaths and DALYs due to IHD in WCBA are still on the rise, reflecting an increasing incidence despite advancements in healthcare. While IHD is commonly regarded as a disease primarily affecting older adults, particularly men, the growing incidence of IHD among WCBA highlights the importance of considering gender- and age-specific factors when addressing cardiovascular health (1). Furthermore, the data reveal that MIR for IHD are significantly higher among younger women, especially in low- and middle-SDI regions, suggesting that WCBA may face more severe outcomes from IHD compared to other age groups (22). In 2021, the leading contributors to IHD burden among WCBA were dietary risks, high LDL cholesterol, high systolic blood pressure, air pollution, and high BMI, emphasizing the importance of addressing both lifestyle and metabolic determinants.

### 4.2 Temporal trends and global patterns

A key finding of this study is the significant regional variation in the trends of ischemic heart disease (IHD). While high-income regions, such as North America, saw reductions in ASIR, other regions, particularly in East Asia and Eastern Europe, experienced concerning increases in IHD incidence (23, 24). In addition, the disparities in age-standardized mortality and DALY rates across regions underscore the need for region-specific approaches to address the burden of IHD. In high-SDI regions, where healthcare access is more robust, the risk of premature death from IHD among WCBA is mitigated by better prevention and treatment options. In contrast, lower-SDI regions with limited healthcare infrastructure continue to face significantly higher mortality and disability from IHD, particularly among young women (25). These regional disparities emphasize the importance of tailoring interventions to reduce the cardiovascular burden among WCBA in different contexts (26).

The inverted U-shaped relationship between the burden of IHD and the SDI highlights critical implications for middle-SDI countries, which are often undergoing rapid socioeconomic and epidemiological transitions. These nations frequently face a dual burden: increasing exposure to lifestyle-related risk factors while still lacking robust health systems capable of providing early screening, prevention, and treatment for cardiovascular conditions (11, 27, 28). Women of childbearing age are particularly vulnerable in these contexts due to lower rates of cardiovascular risk assessment, limited awareness of atypical IHD symptoms in women, and competing priorities in reproductive health services (2, 13, 26). Moreover, many middle-SDI countries are in the midst of urbanization and nutritional transitions that promote obesogenic environments without the parallel development of health-promoting infrastructure (27, 29). These dynamics contribute to both rising incidence and sustained high mortality and DALY rates for IHD in WCBA. As such, policy efforts in these settings must prioritize investment in gender-sensitive primary healthcare, public health campaigns tailored to women's cardiovascular risk, and integrated chronic disease management strategies to mitigate the escalating burden of IHD (3, 30, 31).

### 4.3 Regional and national disparities

While rising SDI is associated with higher-than-expected rates of IHD in some regions, particularly North Africa and the Middle East, the relationship is not straightforward. The observed turning point around an SDI value of approximately 0.45 should be interpreted as an empirical peak derived from the LOWESS-smoothed association rather than a strict epidemiological threshold. This pattern is consistent with the theory of epidemiological and socioeconomic transition, whereby middle-SDI countries often undergo rapid urbanization and lifestyle changes that increase exposure to cardiometabolic risks, including unhealthy diets, obesity, hypertension, and diabetes. During this transitional stage, health systems may not yet have sufficient capacity for effective cardiovascular prevention, early diagnosis, and long-term risk-factor management, resulting in rising incidence and sustained burden of IHD. As SDI increases further, improvements in health system infrastructure, access to essential medicines, population-level prevention strategies (e.g., tobacco control, hypertension management), and greater awareness of cardiovascular risk may contribute to declining mortality and DALY rates despite continued exposure to some metabolic risks. Therefore, the inverted U-shaped relationship likely reflects the combined effects of increasing risk-factor prevalence during development followed by enhanced healthcare delivery and chronic disease control in higher-SDI settings.

The high burden observed in North Africa and the Middle East by considering regional factors such as persistent socio-political instability, limited integration of specialized care for gynecological disorders, and cultural stigmas surrounding reproductive and gynecological health, which may delay diagnosis and treatment (32, 33). Additionally, the rising prevalence of metabolic risk factors, such as obesity and diabetes—particularly among women in North Africa and the Middle East—may also contribute to the elevated burden (34). Moreover, the disproportionately high MIR in Oceania may be attributed to disparities in healthcare access, particularly in remote island communities (35). Indigenous populations in Oceania often face systemic barriers to timely diagnosis and care, compounded by limited availability of specialized gynecological services (36). Environmental and lifestyle factors, including higher rates of tobacco use and lower socioeconomic status, may further exacerbate outcomes (35). This emphasizes the urgent need for targeted cardiovascular health interventions, especially for WCBA, who may face limited access to preventive care, diagnostic services, and treatment options (28). Specifically, first, strengthening primary healthcare systems is critical to enable the early detection and continuous management of key cardiovascular risk factors, including hypertension, diabetes, and high BMI (37). Second, integrating cardiovascular risk assessments into routine maternal and reproductive health services offers a unique opportunity to identify at-risk women during frequent healthcare interactions (38, 39). Third, community-based health promotion initiatives should be scaled up to enhance awareness of modifiable risk factors and promote behavior change, with a particular focus on reaching rural and underserved populations (40). Finally, improving access to essential diagnostics and medications can be facilitated through task-shifting strategies, where trained community health workers help deliver preventive and therapeutic services in areas with limited physician availability (25, 41).

### 4.4 Risk factors and attributable burden

In 2021, 90.00% (154,982/172,204) of deaths and 87.90% (7,661,830/8,712,835) of DALYs from IHD among WCBA were attributed to potentially modifiable risk factors. It is important to note that the risk-attributable fractions reported in this study are derived from the GBD comparative risk assessment framework and reflect modeled counterfactual scenarios rather than directly observed causal effects. Specifically, these estimates represent the proportion of IHD burden that could theoretically be reduced if population exposure to a given risk factor were shifted toward a theoretical minimum risk exposure level, based on published relative risks and integrated modeling assumptions. Therefore, these findings should be interpreted as indicating potential prevention priorities at the population level rather than establishing individual-level causality.

Using the GBD comparative risk assessment framework, we found that dietary risks were the largest attributable contributor to IHD deaths and DALYs globally, followed by high LDL cholesterol and high systolic blood pressure. In addition, air pollution and high BMI emerged as major contributors, particularly in low- and middle-SDI settings and in regions undergoing rapid epidemiological transition. These findings highlight areas where preventive efforts could be prioritized, although the attributable fractions represent modeled estimates rather than direct causal effects. The significant roles of diet and high BMI are consistent with previous research, which has shown that lifestyle factors, such as unhealthy eating habits, physical inactivity, and obesity, are driving increases in IHD incidence and mortality across various age groups and genders (14, 42). Notably, the data reveal that the contribution of high BMI to IHD-related deaths has significantly increased over the past three decades. In high-SDI regions, where obesity rates are higher, BMI was responsible for a substantial proportion of IHD-related deaths. This finding highlights the critical need to address obesity as a key risk factor in cardiovascular disease prevention (29). Although high-SDI countries generally benefit from advanced healthcare systems, rising rates of obesity and hypertension—driven by sedentary lifestyles and poor nutrition—are diminishing these advantages (43). Our findings are in line with previous studies documenting the growing cardiovascular burden associated with the obesity epidemic, particularly among young and middle-aged women (44, 45). Our findings are consistent with previous global and regional evidence indicating that metabolic risk factors, particularly high systolic blood pressure and elevated LDL cholesterol, remain among the strongest contributors to IHD burden (46). Large-scale analyses from the GBD framework and other cardiovascular epidemiology studies have repeatedly identified hypertension as a leading modifiable cause of premature cardiovascular mortality, while high LDL cholesterol plays a central role in atherosclerotic disease progression and ischemic events (47, 48). The prominent contribution of these risks in our WCBA-specific analysis underscores that traditional cardiometabolic drivers remain highly relevant even in younger female populations. These results align with prior studies demonstrating that effective blood pressure control and lipid-lowering interventions are among the most impactful strategies for reducing global IHD burden.

We also observed that metabolic risks such as high BMI and high blood pressure exhibit a positive association with SDI, contrasting with environmental risks like air pollution, which tend to decline as development progresses. This divergence highlights the need for context-specific prevention strategies that go beyond clinical care. In high-SDI settings, public health policies must complement healthcare infrastructure by addressing lifestyle-related risk factors through structural interventions, such as promoting healthier food environments and active transportation. The shifting burden toward metabolic risk factors reinforces the importance of integrated strategies targeting clusters of related conditions (46). Given the association between high BMI and metabolic risk factors—such as hypertension, high cholesterol, and diabetes, often exacerbated by poor dietary habits—Interventions addressing these factors may help reduce the future burden of IHD, particularly if implemented alongside broader strategies targeting cardiovascular prevention in WCBA (30). Additionally, air pollution, non-optimal temperatures, and low physical activity were identified as major contributors to IHD deaths, particularly in regions like South Asia, Southeast Asia, and Oceania. The impact of environmental factors on cardiovascular health is increasingly recognized, and this study reinforces the importance of adopting integrated approaches that combine individual lifestyle modifications with broader environmental health interventions (49). Addressing these factors, especially in urban areas with high pollution levels and extreme weather conditions, could help reduce IHD incidence and improve the quality of life for WCBA.

Importantly, WCBA represent a distinct population in whom female-specific and reproductive factors may further modify cardiovascular risk. Pregnancy-related complications, including preeclampsia, gestational hypertension, and gestational diabetes, have been increasingly recognized as independent predictors of future ischemic heart disease and long-term cardiometabolic dysfunction (50–52). These conditions may accelerate vascular injury and contribute to earlier onset of IHD among women. In addition, hormonal changes across the reproductive life course, use of hormonal contraceptives, and premature ovarian dysfunction may also influence lipid metabolism, blood pressure regulation, and inflammatory pathways (53, 54). Although these sex-specific factors are not explicitly quantified within the current GBD risk attribution framework, they remain highly relevant for interpreting the growing burden of IHD among WCBA. Future studies should further integrate reproductive history and pregnancy-related cardiovascular risk into burden assessments to improve prevention strategies tailored to women across the life course.

### 4.5 Implications for public health policy

This study emphasizes the urgent need for public health interventions and policy changes to reduce the burden of IHD among WCBA, particularly in regions experiencing rising incidence. Given the unique health challenges faced by WCBA, including the long-term cardiovascular effects of pregnancy-related conditions such as gestational diabetes and preeclampsia, it is crucial to integrate women's cardiovascular health into both national and global health agendas (55). These efforts should focus on promoting healthier lifestyles, improving access to preventive care, and ensuring the early identification of women at risk for IHD. The findings from this study have several important public health implications. First, the high proportion of IHD-related deaths and DALYs attributable to modifiable risk factors underscores the urgent need for early, population-wide preventive strategies (40). Interventions should target key behavioral and metabolic risks such as high BMI, poor diet, hypertension, and tobacco use. Tailored public health campaigns and community-based programs promoting healthy eating, physical activity, and smoking cessation could substantially reduce cardiovascular risk in this vulnerable group (3, 31). Second, integrating cardiovascular risk screening into existing maternal and reproductive health services could enable earlier identification and management of women at risk for IHD (38). As WCBA often interact with the healthcare system during pregnancy and postpartum periods, these touchpoints present valuable opportunities to assess cardiovascular risk factors, provide counseling, and initiate timely interventions (39). Third, addressing the burden of IHD among WCBA, particular in resource-limited settings, requires strengthening primary healthcare systems, investing in workforce training, and improving access to essential diagnostics and medications (37, 41). Furthermore, the increasing burden of IHD among WCBA highlights the importance of addressing broader environmental and social determinants of health, including air pollution, socioeconomic inequalities, education, and access to healthcare (56, 57). Tackling these disparities is essential for reducing the cardiovascular burden in this population, especially in low- and middle-income countries.

### 4.6 Limitations

While this study provides valuable insights into the global burden of IHD in WCBA, it has several limitations. Firstly, the data are based on estimates from the GBD 2021 study, which relies on available data from a variety of sources. The quality and completeness of these data may vary across regions, particularly in low- and middle-income countries where vital statistics are often incomplete. In regions with weak health surveillance systems underreporting and limited data quality may affect the accuracy of the modeled estimates. These limitations may lead to under- or over-estimation of the true burden of IHD and associated risk factors among WCBA. In addition, the present analysis is based on the GBD 2021 study cycle; as GBD estimates are periodically updated with newly available data and methodological refinements (e.g., in more recent releases such as GBD 2023), some numerical values of burden metrics and attributable risk fractions may be slightly revised in future iterations. However, the overall patterns and conclusions are expected to remain robust. Secondly, this study is based on population-level data and therefore subject to the ecological fallacy. The associations observed between IHD burden and attributable risk factors reflect population-level trends and cannot be interpreted as causal relationships at the individual level. Additionally, this study did not examine the role of genetic and hormonal factors, which may also contribute to the risk of IHD in women. Future research should focus on understanding the complex interactions between genetic, hormonal, and environmental factors in the development of IHD among WCBA, and explore how these factors contribute to the observed regional and sex-specific disparities in disease burden. Furthermore, temporal trends in IHD burden may not always follow a constant linear pattern over the entire 1990–2021 period. Although EAPC is widely used in GBD-based analyses to summarize average long-term changes and ensure comparability across countries and regions, segmented (joinpoint) regression approaches may better capture potential inflection points or nonlinear trend shifts. WCBA (15–49 years) comprise a heterogeneous population, and cardiovascular risk factors, clinical presentation, and outcomes may differ substantially between adolescents, young adults, and women in their late reproductive years (9). While our analysis provides an overall assessment of IHD burden across the reproductive life course, more granular stratified analyses (e.g., 15–19, 20–34, and 35–49 years) may offer additional insights into age-specific risk trajectories and should be explored in future studies.

## 5 Conclusions

In conclusion, this study provides compelling evidence of the rising burden of ischemic heart disease among women of childbearing age globally. Addressing this burden requires comprehensive, gender-specific strategies that focus on prevention, early diagnosis, and the management of modifiable risk factors, as well as broader efforts to improve access to healthcare and address social determinants of health.

## Data Availability

The original contributions presented in the study are included in the article/Supplementary Material, further inquiries can be directed to the corresponding author.

## References

[1] Roth GA Mensah GA Johnson CO Addolorato G Ammirati E Baddour LM Global burden of cardiovascular diseases and risk factors, 1990–2019: update from the GBD 2019 study. J Am Coll Cardiol. (2020) 76(25):2982–3021. 10.1016/j.jacc.2020.11.010 33309175 PMC7755038

[2] Minissian MB Mehta PK Hayes SN Park K Wei J Bairey Merz CN Ischemic heart disease in young women: JACC review topic of the week. J Am Coll Cardiol. (2022) 80(10):1014–22. 10.1016/j.jacc.2022.01.057 PMC9847245 36049799

[3] Wang LY-T Chiang GSH Wee CF Chan SWK Lau JXX Taeihagh A. Preventing ischemic heart disease in women: a systematic review of global directives and policies. NPJ Women’s Health. (2024) 2(1):36. 10.1038/s44294-024-00040-0

[4] Yuan R Tong Z Chen JX Wang Y Zhou YF. Global burden of ischemic heart disease in adolescents and young adults, 1990–2019. Am J Prev Med. (2024) 66(5):751–9. 10.1016/j.amepre.2023.12.009 38104848

[5] Arora S Stouffer GA Kucharska-Newton AM Qamar A Vaduganathan M Pandey A Twenty year trends and sex differences in young adults hospitalized with acute myocardial infarction. Circulation. (2019) 139(8):1047–56. 10.1161/CIRCULATIONAHA.118.037137 30586725 PMC6380926

[6] Mehta LS Beckie TM DeVon HA Grines CL Krumholz HM Johnson MN Acute myocardial infarction in women: a scientific statement from the American Heart Association. Circulation. (2016) 133(9):916–47. 10.1161/CIR.0000000000000351 26811316

[7] O'Kelly AC Michos ED Shufelt CL Vermunt JV Minissian MB Quesada O Pregnancy and reproductive risk factors for cardiovascular disease in women. Circ Res. (2022) 130(4):652–72. 10.1161/CIRCRESAHA.121.319895 35175837 PMC8870397

[8] Melchiorre K Sharma R Thilaganathan B. Cardiovascular implications in preeclampsia: an overview. Circulation. (2014) 130(8):703–14. 10.1161/CIRCULATIONAHA.113.003664 25135127

[9] Scott J Agarwala A Baker-Smith CM Feinstein MJ Jakubowski K Kaar J Cardiovascular health in the transition from adolescence to emerging adulthood: a scientific statement from the American Heart Association. J Am Heart Assoc. (2025) 14(9):e039239. 10.1161/JAHA.124.039239 40135400 PMC12184556

[10] Yusuf S Joseph P Rangarajan S Islam S Mente A Hystad P Modifiable risk factors, cardiovascular disease, and mortality in 155 722 individuals from 21 high-income, middle-income, and low-income countries (PURE): a prospective cohort study. Lancet. (2020) 395(10226):795–808. 10.1016/S0140-6736(19)32008-2 31492503 PMC8006904

[11] GBD 2021 Risk Factors Collaborators.: global burden and strength of evidence for 88 risk factors in 204 countries and 811 subnational locations, 1990–2021: a systematic analysis for the global burden of disease study 2021. Lancet. (2024) 403(10440):2162–203. 10.1016/S0140-6736(24)00933-4 38762324 PMC11120204

[12] Vogel B Acevedo M Appelman Y Bairey Merz CN Chieffo A Figtree GA The lancet women and cardiovascular disease commission: reducing the global burden by 2030. Lancet. (2021) 397(10292):2385–438. 10.1016/S0140-6736(21)00684-X 34010613

[13] Mattina DJ Honigberg MC Mahmoud Z Joynt Maddox KE. Strategies for overcoming barriers in access to cardiovascular care for women. Circ Res. (2025) 136(6):628–41. 10.1161/CIRCRESAHA.124.325544 40080535 PMC11921933

[14] Shu T Tang M He B Liu X Han Y Liu C Assessing global, regional, and national time trends and associated risk factors of the mortality in ischemic heart disease through global burden of disease 2019 study: population-based study. JMIR Public Health Surveill. (2024) 10:e46821. 10.2196/46821 38265846 PMC10851120

[15] NCD Countdown 2030 collaborators. NCD Countdown 2030: pathways to achieving sustainable development goal target 3.4. Lancet. (2020) 396(10255):918–34. 10.1016/S0140-6736(20)31761-X 32891217 PMC7470795

[16] GBD 2021 Diseases and Injuries Collaborators. Global incidence, prevalence, years lived with disability (YLDs), disability-adjusted life-years (DALYs), and healthy life expectancy (HALE) for 371 diseases and injuries in 204 countries and territories and 811 subnational locations, 1990–2021: a systematic analysis for the global burden of disease study 2021. Lancet. (2024) 403(10440):2133–61. 10.1016/S0140-6736(24)00757-8 38642570 PMC11122111

[17] Murray CJL. The global burden of disease study at 30 years. Nat Med. (2022) 28(10):2019–26. 10.1038/s41591-022-01990-1 36216939

[18] GBD 2021 Demographics Collaborators. Global age-sex-specific mortality, life expectancy, and population estimates in 204 countries and territories and 811 subnational locations, 1950–2021, and the impact of the COVID-19 pandemic: a comprehensive demographic analysis for the global burden of disease study 2021. Lancet. (2024) 403(10440):1989–2056. 10.1016/S0140-6736(24)00476-8 38484753 PMC11126395

[19] Fay MP Feuer EJ. Confidence intervals for directly standardized rates: a method based on the gamma distribution. Stat Med. (1997) 16(7):791–801. 10.1002/(SICI)1097-0258(19970415)16:7<791::AID-SIM500>3.0.CO;2-# 9131766 10.1002/(sici)1097-0258(19970415)16:7<791::aid-sim500>3.0.co;2-#

[20] Hankey BF Ries LA Kosary CL Feuer EJ Merrill RM Clegg LX Partitioning linear trends in age-adjusted rates. Cancer Causes Control. (2000) 11(1):31–5. 10.1023/A:1008953201688 10680727

[21] Cleveland WS. Robust locally weighted regression and smoothing scatterplots. J Am Stat Assoc. (1979) 74(368):829–36. 10.1080/01621459.1979.10481038

[22] DeFilippis EM Collins BL Singh A Biery DW Fatima A Qamar A Women who experience a myocardial infarction at a young age have worse outcomes compared with men: the mass general brigham YOUNG-MI registry. Eur Heart J. (2020) 41(42):4127–37. 10.1093/eurheartj/ehaa662 33049774 PMC7700756

[23] Meng R Wang W Huang T Zhan S. Tackling social and behavioural risk factors for cardiovascular diseases in Chinese women. Br Med J. (2024) 386:e078638. 10.1136/bmj-2023-078638 39214527 PMC11359725

[24] Cenko E Manfrini O Fabin N Dorobantu M Kedev S Milicic D Clinical determinants of ischemic heart disease in Eastern Europe. Lancet Reg Health Eur. (2023) 33:100698. 10.1016/j.lanepe.2023.100698 37954000 PMC10636265

[25] Schwalm JD McKee M Huffman MD Yusuf S. Resource effective strategies to prevent and treat cardiovascular disease. Circulation. (2016) 133(8):742–55. 10.1161/CIRCULATIONAHA.115.008721 26903017 PMC4766731

[26] Nabel EG. Heart disease prevention in young women: sounding an alarm. Circulation. (2015) 132(11):989–91. 10.1161/CIRCULATIONAHA.115.018352 26302760

[27] Miranda JJ Barrientos-Gutiérrez T Corvalan C Hyder AA Lazo-Porras M Oni T Understanding the rise of cardiometabolic diseases in low- and middle-income countries. Nat Med. (2019) 25(11):1667–79. 10.1038/s41591-019-0644-7 31700182

[28] Wang H Yu X Guo J Ma S Liu Y Hu Y Burden of cardiovascular disease among the western Pacific region and its association with human resources for health, 1990–2021: a systematic analysis of the global burden of disease study 2021. Lancet Reg Health West Pac. (2024) 51:101195. 10.1016/j.lanwpc.2024.101195 39286450 PMC11404088

[29] Manrique-Acevedo C Chinnakotla B Padilla J Martinez-Lemus LA Gozal D. Obesity and cardiovascular disease in women. Int J Obes (Lond). (2020) 44(6):1210–26. 10.1038/s41366-020-0548-0 32066824 PMC7478041

[30] Li Y Sun Y Wu H Yang P Huang X Zhang L Metabolic syndromes increase significantly with the accumulation of bad dietary habits. J Nutr Health Aging. (2024) 28(2):100017. 10.1016/j.jnha.2023.100017 38388115 PMC12877254

[31] Vervoort D Wang R Li G Filbey L Maduka O Brewer LC Addressing the global burden of cardiovascular disease in women: JACC state-of-the-art review. J Am Coll Cardiol. (2024) 83(25):2690–707. 10.1016/j.jacc.2024.04.028 38897679

[32] Saleh S Fouad FM. Political economy of health in fragile and conflict-affected regions in the Middle East and north Africa region. J Glob Health. (2022) 12:01003. 10.7189/jogh.12.01003 35959965 PMC9373566

[33] Oraby D. Determinants of knowledge in relation to sexual and reproductive health of adolescents in the Middle East and north Africa region. In: Barakat C Dghaim R Al Anouti F, editors. Adolescent Health in the Middle East and North Africa: An Epidemiological Perspective. Cham: Springer International Publishing (2022). p. 95–104.

[34] Azizi F Hadaegh F Hosseinpanah F Mirmiran P Amouzegar A Abdi H Metabolic health in the Middle East and north Africa. Lancet Diabetes Endocrinol. (2019) 7(11):866–79. 10.1016/S2213-8587(19)30179-2 31422063

[35] Venketasubramanian N. Stroke epidemiology in oceania: a review. Neuroepidemiology. (2021):1–10. 10.1159/000512972 33601397

[36] Horwood PF Tarantola A Goarant C Matsui M Klement E Umezaki M Health challenges of the Pacific region: insights from history, geography, social determinants, genetics, and the microbiome. Front Immunol. (2019) 10:2184. 10.3389/fimmu.2019.02184 31572391 PMC6753857

[37] Stenberg K Hanssen O Bertram M Brindley C Meshreky A Barkley S Guide posts for investment in primary health care and projected resource needs in 67 low-income and middle-income countries: a modelling study. Lancet Glob Health. (2019) 7(11):e1500–10. 10.1016/S2214-109X(19)30416-4 31564629 PMC7024989

[38] Grandi SM. Cardiovascular risk screening in women with pregnancy complications: the need for integrative strategies. J Womens Health (Larchmt). (2021) 30(3):285–6. 10.1089/jwh.2020.8716 32780667 PMC7957367

[39] Adedinsewo DA Pollak AW Phillips SD Smith TL Svatikova A Hayes SN Cardiovascular disease screening in women: leveraging artificial intelligence and digital tools. Circ Res. (2022) 130(4):673–90. 10.1161/CIRCRESAHA.121.319876 35175849 PMC8889564

[40] Gupta R Wood DA. Primary prevention of ischaemic heart disease: populations, individuals, and health professionals. Lancet. (2019) 394(10199):685–96. 10.1016/S0140-6736(19)31893-8 31448740

[41] Marthias T Anindya K Saputri NS Putri LP Atun R Lee JT. Effective coverage for reproductive, maternal, neonatal and newborn health: an analysis of geographical and socioeconomic inequalities in 39 low- and middle-income countries. BMJ Glob Health. (2025) 10(2):e016549. 10.1136/bmjgh-2024-016549 39961692 PMC11836785

[42] Wang C Sun Y Jiang D Wang C Liu S. Risk-Attributable burden of ischemic heart disease in 137 low- and middle-income countries from 2000 to 2019. J Am Heart Assoc. (2021) 10(19):e021024. 10.1161/JAHA.121.021024 34585592 PMC8649139

[43] Evans M de Courcy J de Laguiche E Faurby M Haase CL Matthiessen KS Obesity-related complications, healthcare resource use and weight loss strategies in six European countries: the RESOURCE survey. Int J Obes (Lond). (2023) 47(8):750–7. 10.1038/s41366-023-01325-1 37258646 PMC10359184

[44] Virani SS Alonso A Aparicio HJ Benjamin EJ Bittencourt MS Callaway CW Heart disease and stroke statistics-2021 update: a report from the American Heart Association. Circulation. (2021) 143(8):e254–743. 10.1161/CIR.0000000000000950 33501848 PMC13036842

[45] Dikaiou P Björck L Adiels M Lundberg CE Mandalenakis Z Manhem K Obesity, overweight and risk for cardiovascular disease and mortality in young women. Eur J Prev Cardiol. (2021) 28(12):1351–9. 10.1177/2047487320908983 34647583

[46] Wang W Hu M Liu H Zhang X Li H Zhou F Global burden of disease study 2019 suggests that metabolic risk factors are the leading drivers of the burden of ischemic heart disease. Cell Metab. (2021) 33(10):1943–56. e1942. 10.1016/j.cmet.2021.08.005 34478633

[47] Mihaylova B Emberson J Blackwell L Keech A Simes J Barnes EH The effects of lowering LDL cholesterol with statin therapy in people at low risk of vascular disease: meta-analysis of individual data from 27 randomised trials. Lancet. (2012) 380(9841):581–90. 10.1016/S0140-6736(12)60367-5 22607822 PMC3437972

[48] Blood Pressure Lowering Treatment Trialists’ Collaboration. Pharmacological blood pressure lowering for primary and secondary prevention of cardiovascular disease across different levels of blood pressure: an individual participant-level data meta-analysis. Lancet. (2021) 397(10285):1625–36. 10.1016/S0140-6736(21)00590-0 33933205 PMC8102467

[49] Blaustein JR Quisel MJ Hamburg NM Wittkopp S. Environmental impacts on cardiovascular health and biology: an overview. Circ Res. (2024) 134(9):1048–60. 10.1161/CIRCRESAHA.123.323613 38662864 PMC11058466

[50] Lo CCW Lo ACQ Leow SH Fisher G Corker B Batho O: future cardiovascular disease risk for women with gestational hypertension: a systematic review and meta-analysis. J Am Heart Assoc. (2020) 9(13):e013991. 10.1161/JAHA.119.013991 32578465 PMC7670531

[51] Inversetti A Pivato CA Cristodoro M Latini AC Condorelli G Di Simone N Update on long-term cardiovascular risk after pre-eclampsia: a systematic review and meta-analysis. Eur Heart J Qual Care Clin Outcomes. (2024) 10(1):4–13. 10.1093/ehjqcco/qcad065 37974053

[52] Yu Y Soohoo M Sørensen HT Li J Arah OA. Gestational diabetes Mellitus and the risks of overall and type-specific cardiovascular diseases: a population- and sibling-matched cohort study. Diabetes Care. (2022) 45(1):151–9. 10.2337/dc21-1018 34764208 PMC8753767

[53] Honigberg MC Zekavat SM Aragam K Finneran P Klarin D Bhatt DL Association of premature natural and surgical menopause with incident cardiovascular disease. JAMA. (2019) 322(24):2411–21. 10.1001/jama.2019.19191 31738818 PMC7231649

[54] Yonis H Løkkegaard E Kragholm K Granger CB Møller AL Mørch LS Stroke and myocardial infarction with contemporary hormonal contraception: real-world, nationwide, prospective cohort study. Br Med J. (2025) 388:e082801. 10.1136/bmj-2024-082801 39938934 PMC11816856

[55] Lane-Cordova AD Khan SS Grobman WA Greenland P Shah SJ. Long-Term cardiovascular risks associated with adverse pregnancy outcomes: JACC review topic of the week. J Am Coll Cardiol. (2019) 73(16):2106–16. 10.1016/j.jacc.2018.12.092 31023435

[56] Liang F Liu F Huang K Yang X Li J Xiao Q Long-Term exposure to fine particulate matter and cardiovascular disease in China. J Am Coll Cardiol. (2020) 75(7):707–17. 10.1016/j.jacc.2019.12.031 32081278

[57] Zheng Y Wen X Bian J Zhao J Lipkind HS Hu H. Racial, ethnic, and geographic disparities in cardiovascular health among women of childbearing age in the United States. J Am Heart Assoc. (2021) 10(17):e020138. 10.1161/JAHA.120.020138 34431309 PMC8649299

