# Supplementary material for: Global burden and trend of ischemic heart disease and its attributable risk factors among women of childbearing age from 1990 to 2021
Source: Front Cardiovasc Med. 2026 Mar 18;13:1695600. doi: 10.3389/fcvm.2026.1695600 (PMC13038546; doi:10.3389/fcvm.2026.1695600)
Supplement: Supplementary file 1 [file Datasheet1.pdf]

## Supplementary Methods

## Case definition

Ischemic heart disease (IHD) is a disease that limits the supply of blood to the heart. IHD is typically due to the narrowing of the coronary arteries, usually due to atherosclerosis, which limits blood flow. GBD estimates IHD as the aggregate of discrete sequelae, consisting of myocardial infarction (heart attacks), angina (stable IHD manifesting as chest pain), or ischemic cardiomyopathy (heart failure due to IHD). The GBD 2021 modelled prevalence and incidence of acute myocardial infarction (MI) as well as the prevalence of chronic IHD.

(1) Acute myocardial infarction (MI): Definite and possible MI according to the fourth universal definition of myocardial infarction:

a. When there is clinical evidence of myocardial necrosis in a clinical setting consistent with myocardial ischaemia or

b. Detection of a rise and/or fall of cardiac biomarker values and with at least one of the following: i) symptoms of ischemia, ii) new or presumed new ST-segment-T wave changes or new left bundle branch block, iii) development of pathological Q waves in the ECG, iv) imaging evidence of new loss of viable myocardium or new regional wall motion abnormality, or v) identification of an intracoronary thrombus by angiography or autopsy.

c. Sudden (abrupt) unexplained cardiac death, involving cardiac arrest or no evidence of a non-coronary cause of death. The prevalence of MI is considered to last from the onset of the event to 28 days after the event and is divided into an acute phase (0–2 days) and subacute phase (3–28 days). The GBD also included unstable angina when reported separately as specified in the fourth universal definition.

(2) Chronic IHD

a. Stable angina: clinically diagnosed stable exertional angina pectoris or definite angina pectoris according to the Rose Angina Questionnaire, physician diagnosis, or taking nitrate medication for the relief of chest pain.

b. Asymptomatic ischaemic heart disease following myocardial infarction; survival to 28 days following incident MI. The GBD study does not use estimates based on ECG evidence for prior MI, due to its limited specificity and sensitivity.

## Input data and Modelling strategy

For IHD, the input data were derived from multiple sources, including vital registration systems, verbal autopsy reports, health surveys, hospital records, and published literature. These data sources were rigorously

assessed for completeness, quality, and internal consistency before being incorporated into the modeling process. Cause-specific mortality due to IHD was modeled using the Cause of Death Ensemble model (CODEm), a highly flexible, predictive modeling tool that tests a wide range of models and covariate combinations to select the best-performing ensemble model based on out-of-sample predictive validity. CODEm estimates age-, sex-, and location-specific death rates while addressing data sparsity and inconsistencies across regions. For non-fatal estimates such as incidence, prevalence, and years lived with disability (YLDs), the DisMod-MR 2.1 meta-regression tool was employed. This Bayesian compartmental model ensures internal consistency between related epidemiological parameters (i.e., incidence, prevalence, remission, and excess mortality) and allows for borrowing of strength across geographies and time, especially in data-limited settings. IHD was defined based on International Classification of Diseases (ICD) codes, including ICD-10 codes I20-I25.9 and corresponding ICD-9 codes 410-414.9 and V17.3. Data harmonization and adjustments were applied to ensure comparability across data sources and coding systems. To quantify uncertainty, all estimates were based on 1,000 draws from the posterior distribution, with 95% uncertainty intervals (UIs) reported for each metric, reflecting variability due to sampling, model choice, and other sources of uncertainty.

**Risk factors: Population attributable fraction estimation**

The GBD Collaborative employs a rigorous, data-rich methodology based on a counterfactual comparative risk assessment framework to estimate the population attributable fraction (PAF). The primary goal is to determine the proportion of disease or death that could be prevented if exposure to a specific risk factor—or set of risk factors—were reduced to a Theoretical Minimum Risk Exposure Level (TMREL), defined as the exposure level associated with the lowest possible risk, though not necessarily zero. The calculation involves comparing the current burden of disease under existing exposure levels with the hypothetical burden if the entire population were exposed to the TMREL.

In the GBD 2021, there are 11 risk factors associated with IHD: (1) Environmental/Occupational Risks: Air pollution, non-optimal temperature, and other environmental risks; (2) Behavioral Risks: Dietary risks, tobacco use, and low physical activity; (3) Metabolic Risks: High body mass index (BMI), high systolic blood pressure, high fasting plasma glucose, high LDL cholesterol, and kidney dysfunction. The corresponding TMREL of each risk factor are presented in the Supplementary appendix 1 of the GBD 2021 Risk Factors

Collaborators capstone paper (GBD 2021 Risk Factors Collaborators: Global burden and strength of evidence for 88 risk factors in 204 countries and 811 subnational locations, 1990-2021: a systematic analysis for the Global Burden of Disease Study 2021. Lancet 2024, 403(10440):2162-2203).

The resulting PAF reflects the proportion of cases or deaths that could be averted under this optimal exposure scenario. While this framework does not imply causality at the individual level, it enables a robust population-level understanding of how much disease burden is attributable to modifiable risk factors. The attributable burden is then estimated by multiplying the total burden of a specific health outcome by its corresponding PAF. This process involves six key steps: (1) identifying valid risk–outcome pairs; (2) estimating relative risks as functions of exposure; (3) assessing exposure distributions within the population; (4) defining the TMREL; (5) calculating the PAF and attributable burden; and (6) accounting for mediation between risk factors to estimate their combined effects. A critical component of the GBD approach is its treatment of uncertainty. Using Bayesian meta-regression models and extensive Monte Carlo simulations, the methodology propagates uncertainty from all inputs—such as relative risks, exposure levels, TMRELs, and baseline disease rates—across thousands of iterations to generate 95% uncertainty intervals (UIs) for each estimate.

This approach is characterized by several strengths: a counterfactual framework anchored in TMREL targets, use of continuous non-linear risk curves, the ability to model multiple interacting risks simultaneously, integration of extensive global datasets, and robust uncertainty quantification. Together, these elements make the GBD PAF methodology a global benchmark for assessing the impact of risk factors on population health.

**Supplementary Table S1. 21 GBD world regions and 204 countries and territories within each region.**

| GBD World Region (n=21) | Countries and territories (n=204) |
|---|---|
| Central Asia | Armenia, Azerbaijan, Georgia, Kazakhstan, Kyrgyzstan, Mongolia, Tajikistan, Turkmenistan, Uzbekistan |
| Central Europe | Albania, Bosnia and Herzegovina, Bulgaria, Croatia, Czech Republic, Hungary, Montenegro, North Macedonia, Poland, Romania, Serbia, Slovakia, Slovenia |
| Eastern Europe | Belarus, Estonia, Latvia, Lithuania, Moldova, Russia, Ukraine |
| Australasia | Australia, New Zealand |
| High-income Asia Pacific | Brunei, Japan, Singapore, South Korea |
| High-income North America | Canada, Greenland, United States |
| Southern Latin America | Argentina, Chile, Uruguay |
| Western Europe | Andorra, Austria, Belgium, Cyprus, Denmark, Finland, France, Germany, Greece, Iceland, Ireland, Israel, Italy, Luxembourg, Malta, Monaco, Netherlands, Norway, Portugal, San Marino, Spain, Sweden, Switzerland, United Kingdom |
| Andean Latin America | Bolivia, Ecuador, Peru |
| Caribbean | Antigua and Barbuda, Bahamas, Barbados, Belize, Bermuda, Cuba, Dominica, Dominican Republic, Grenada, Guyana, Haiti, Jamaica, Puerto Rico, Saint Kitts and Nevis, Saint Lucia, Saint Vincent and the Grenadines, Suriname, Trinidad and Tobago, US Virgin Islands |
| Central Latin America | Colombia, Costa Rica, El Salvador, Guatemala, Honduras, Mexico, Nicaragua, Panama, Venezuela |
| Tropical Latin America | Brazil, Paraguay |
| North Africa and Middle East | Afghanistan, Algeria, Bahrain, Egypt, Iran, Iraq, Jordan, Kuwait, Lebanon, Libya, Morocco, Oman, Palestine, Qatar, Saudi Arabia, Sudan, Syria, Tunisia, Türkiye, United Arab Emirates, Yemen |
| South Asia | Bangladesh, Bhutan, India, Nepal, Pakistan |
| East Asia | China, North Korea, Taiwan (province of China) |
| Oceania | American Samoa, Cook Islands, Federated States of Micronesia, Fiji, Guam, Kiribati, Marshall Islands, Nauru, Niue, Northern Mariana Islands, Palau, Papua New Guinea, Samoa, Solomon Islands, Tokelau, Tonga, Tuvalu, Vanuatu |
| Southeast Asia | Cambodia, Indonesia, Laos, Malaysia, Maldives, Mauritius, Myanmar, Philippines, Seychelles, Sri Lanka, Thailand, Timor-Leste, Vietnam |
| Central sub-Saharan Africa | Angola, Central African Republic, Congo (Brazzaville), Democratic Republic of the Congo, Equatorial Guinea, Gabon |
| Eastern sub-Saharan Africa | Burundi, Comoros, Djibouti, Eritrea, Ethiopia, Kenya, Madagascar, Malawi, Mozambique, Rwanda, Somalia, South Sudan, Tanzania, Uganda, Zambia |
| Southern sub-Saharan Africa | Botswana, eSwatini, Lesotho, Namibia, South Africa, Zimbabwe |
| Western sub-Saharan Africa | Benin, Burkina Faso, Cape Verde, Cameroon, Chad, Côte d'Ivoire, Gambia, Ghana, Guinea, Guinea-Bissau, Liberia, Mali, Mauritania, Niger, Nigeria, São Tomé and Príncipe, Senegal, Sierra Leone, Togo |

**Supplementary Table S2. Socio-demographic Index (SDI) quintiles for 204 countries and territories estimated in GBD 2021**

| SDI Quintile | Locations included based on SDI values in 2021 from GBD 2021 results |
|---|---|
| Low SDI | Afghanistan, Angola, Benin, Burkina Faso, Burundi, Central African Republic, Chad, Côte d'Ivoire, Democratic Republic of the Congo, Eritrea, Ethiopia, Gambia, Guinea, Guinea-Bissau, Haiti, Liberia, Madagascar, Malawi, Mali, Mozambique, Nepal, Niger<br>Papua New Guinea, Rwanda, Senegal, Sierra Leone, Solomon Islands, Somalia, South Sudan, Timor-Leste, Togo, Uganda, United Republic of Tanzania, Yemen |
| Low-middle SDI | Bangladesh, Bhutan, Bolivia (Plurinational State of), Cabo Verde, Cambodia, Cameroon, Comoros, Congo, Democratic People's Republic of Korea, Djibouti, Egypt, El Salvador, Eswatini, Ghana, Guatemala, Honduras, India, Kenya, Kiribati, Kyrgyzstan, Lao People's Democratic Republic, Lesotho, Marshall Islands, Mauritania, Micronesia (Federated States of), Morocco, Myanmar<br>Nicaragua, Nigeria, Pakistan, Samoa, Sao Tome and Principe, Sudan, Tajikistan, Tuvalu, Vanuatu, Venezuela (Bolivarian Republic of), Zambia, Zimbabwe |
| Middle SDI | Algeria, Belize, Botswana, Brazil, Colombia, Cuba, Dominican Republic, Ecuador, Equatorial Guinea, Fiji, Gabon, Grenada, Guyana, Indonesia, Iraq, Jamaica, Maldives, Mexico, Mongolia, Namibia, Nauru, Palestine, Paraguay, Peru, Philippines, Saint Lucia, Saint Vincent and the Grenadines, South Africa, Suriname, Syrian Arab Republic, Thailand, Tokelau, Tonga, Tunisia, Turkmenistan, Uzbekistan, Viet Nam |
| High-middle SDI | Albania, American Samoa, Antigua and Barbuda, Argentina, Armenia, Azerbaijan, Bahrain, Barbados, Belarus, Bosnia and Herzegovina, Bulgaria, Chile, China, Cook Islands, Costa Rica, Croatia, Dominica, Georgia, Greece, Guam, Hungary, Iran (Islamic Republic of), Jordan, Kazakhstan, Lebanon, Libya, Malaysia, Malta, Mauritius, Montenegro, Niue, North Macedonia, Northern Mariana Islands, Oman, Palau, Panama, Portugal, Republic of Moldova, Romania, Saint Kitts and Nevis, Serbia, Seychelles, Spain, Sri Lanka, Trinidad and Tobago, Turkey, Ukraine, Uruguay |
| High SDI | Andorra, Australia, Austria, Bahamas, Belgium, Bermuda, Brunei Darussalam, Canada, Cyprus, Czechia, Denmark, Estonia, Finland, France, Georgia, Germany, Greenland, Iceland, Ireland, Israel, Italy, Japan, Kuwait, Latvia, Lithuania, Luxembourg, Monaco, Netherlands, New Zealand, Norway, Poland, Puerto Rico, Qatar, Republic of Korea, Russian Federation, San Marino, Saudi Arabia, Singapore, Slovakia, Slovenia, Sweden, Switzerland, Taiwan (Province of China), United Arab Emirates, United Kingdom, United States of America, United States Virgin Islands |

**Supplementary Table S3. Mortality-to-incidence ratio of ischemic heart disease among women of childbearing age in 1990 and 2021, and its temporal change over the period 1990–2021**

| Characteristics | Mortality-to-incidence ratio (%), 1990 | Mortality-to-incidence ratio (%), 2021 | Estimated annual percentage changes (95% CI), 1990-2021 |
|---|---|---|---|
| **Global** | 19.45 | 12.99 | -1.43 (-1.48 to -1.38) |
| **Socio-demographic index** | | | |
| High | 13.10 | 8.81 | -1.12 (-1.25 to -0.99) |
| High-middle | 15.20 | 7.15 | -2.86 (-3.1 to -2.62) |
| Middle | 20.41 | 13.08 | -1.52 (-1.65 to -1.39) |
| Low-middle | 24.52 | 16.85 | -1.23 (-1.32 to -1.13) |
| Low | 18.47 | 13.03 | -1.35 (-1.48 to -1.21) |
| **GBD regions** | | | |
| High-income Asia Pacific | 20.61 | 10.28 | -2.37 (-2.53 to -2.21) |
| Central Asia | 25.61 | 15.37 | -2.64 (-3.12 to -2.16) |
| East Asia | 15.64 | 6.60 | -2.73 (-2.9 to -2.57) |
| South Asia | 22.44 | 16.52 | -1.08 (-1.23 to -0.93) |
| Southeast Asia | 53.17 | 39.68 | -1 (-1.07 to -0.93) |
| Australasia | 8.87 | 4.33 | -2.3 (-2.59 to -2.02) |
| Caribbean | 18.94 | 12.84 | -1.06 (-1.26 to -0.86) |
| Central Europe | 15.51 | 6.49 | -3.04 (-3.28 to -2.79) |
| Eastern Europe | 11.74 | 7.65 | -2.56 (-3.22 to -1.89) |
| Western Europe | 15.63 | 6.83 | -2.72 (-2.95 to -2.49) |
| Andean Latin America | 19.63 | 8.22 | -3.12 (-3.5 to -2.74) |
| Central Latin America | 13.59 | 10.26 | -1.01 (-1.41 to -0.61) |
| Southern Latin America | 26.71 | 10.23 | -2.7 (-3.03 to -2.38) |
| Tropical Latin America | 29.82 | 19.45 | -1.86 (-2.02 to -1.71) |
| North Africa and Middle East | 19.36 | 10.84 | -1.92 (-1.98 to -1.86) |
| High-income North America | 10.72 | 12.30 | 1.15 (0.9 to 1.39) |
| Oceania | 40.46 | 36.1 | -0.43 (-0.56 to -0.31) |
| Central Sub-Saharan Africa | 10.64 | 8.59 | -0.75 (-0.83 to -0.67) |
| Eastern Sub-Saharan Africa | 11.65 | 8.50 | -1.3 (-1.4 to -1.19) |
| Southern Sub-Saharan Africa | 10.31 | 7.66 | 0.04 (-0.67 to 0.75) |
| Western Sub-Saharan Africa | 12.91 | 8.91 | -1.28 (-1.45 to -1.12) |

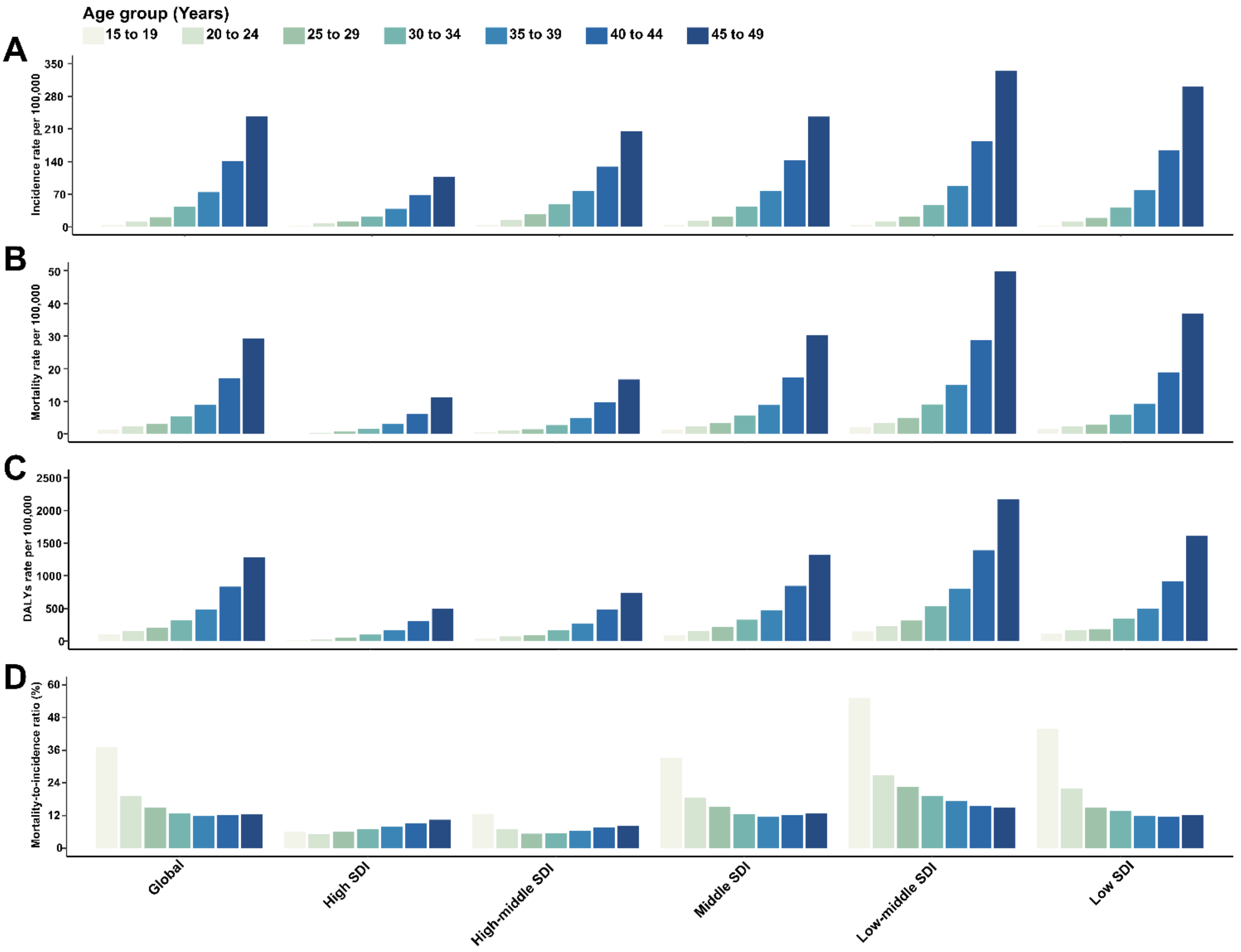


**Supplementary Figure S1.** The burden of ischemic heart disease among women of childbearing age in 2021, globally and across 5 SDI regions, by age group. (A) The age-specific incidence rate of ischemic heart disease. (B) The age-specific mortality rate of ischemic heart disease. (C) The age-specific DALYs rate of ischemic heart disease. (D) The age-specific mortality-to-incidence ratio of ischemic heart disease. DALY=Disability-adjusted life-years; SDI=Socio-demographic index.

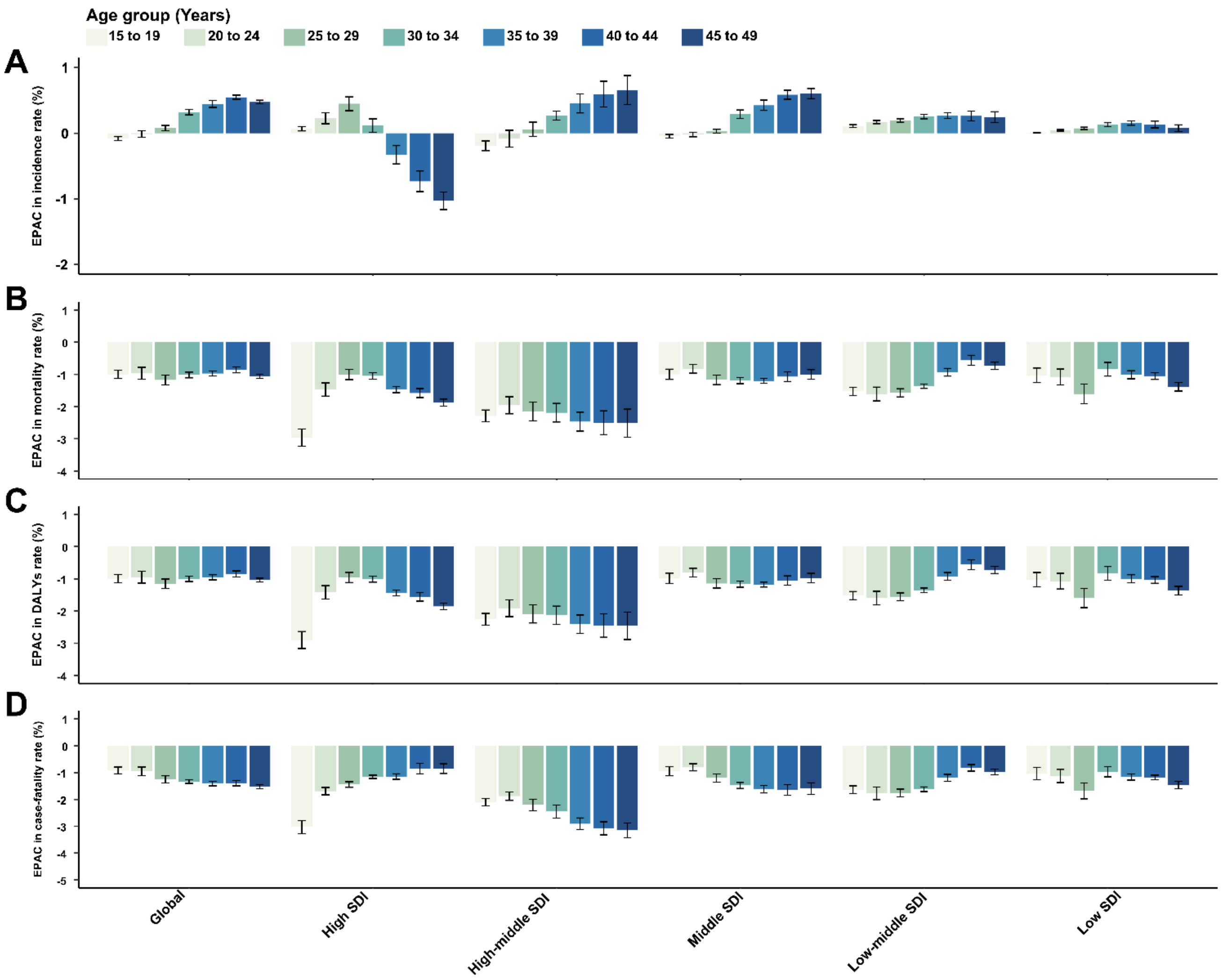


**Supplementary Figure S2.** The estimated annual percentage changes in the (A) incidence rate, (B) mortality rate, (C) DALY rate, and (D) Mortality-to-incidence ratio for ischemic heart disease among women of childbearing age from 1990 to 2021, by age group and SDI. EAPC=Estimated annual percentage changes. DALY=Disability-adjusted life-years;SDI=Socio-demographic index.

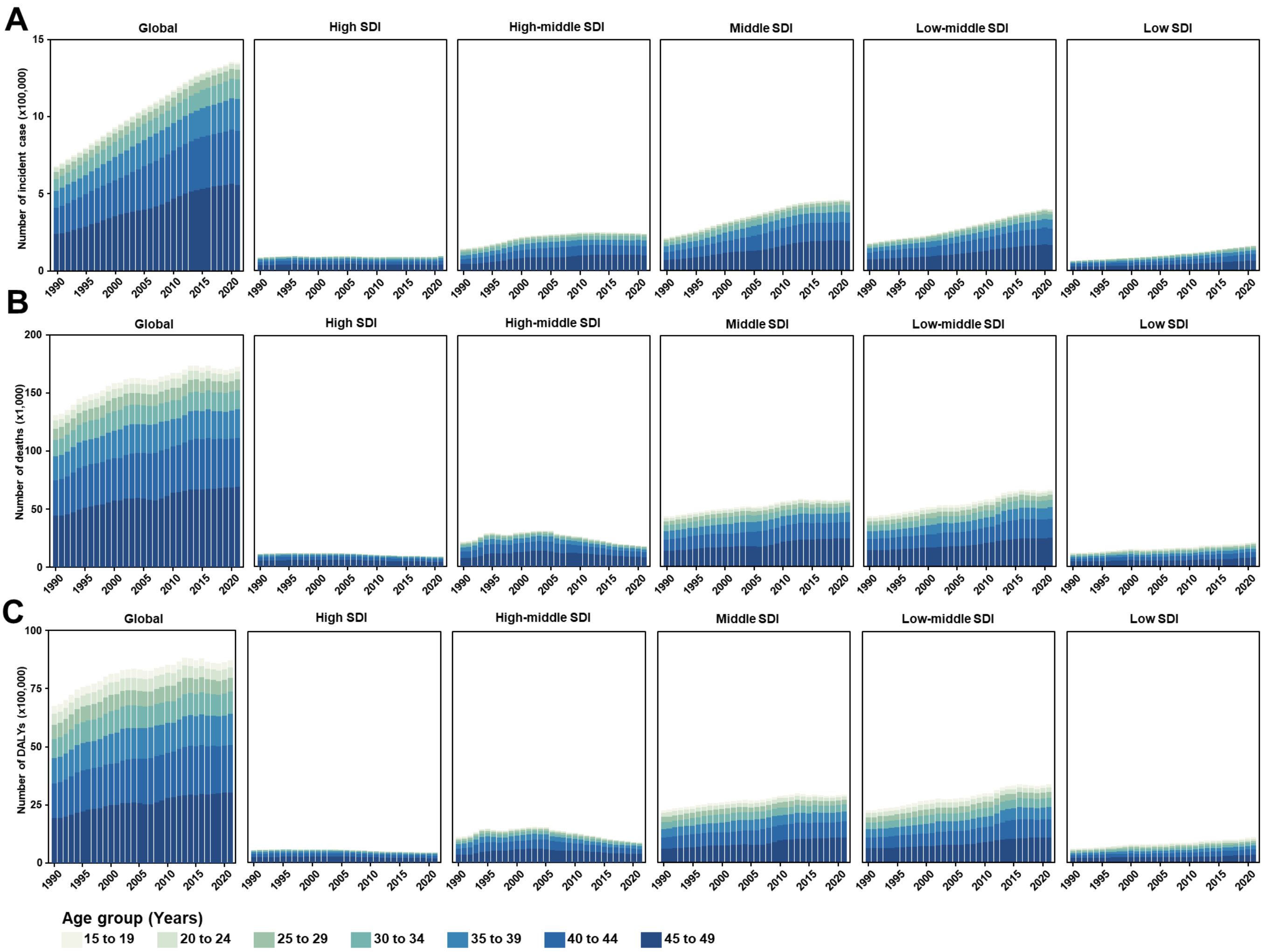


**Supplementary Figure S3.** The number of ischemic heart disease burden among women of childbearing age from 1990 to 2021, globally and across 5 SDI regions, by age group. (A) The number of incident cases of ischemic heart disease. (B) The number of deaths caused by ischemic heart disease. (C) The number of DALYs caused by ischemic heart disease. DALY=Disability-adjusted life-years; SDI=Socio-demographic index.

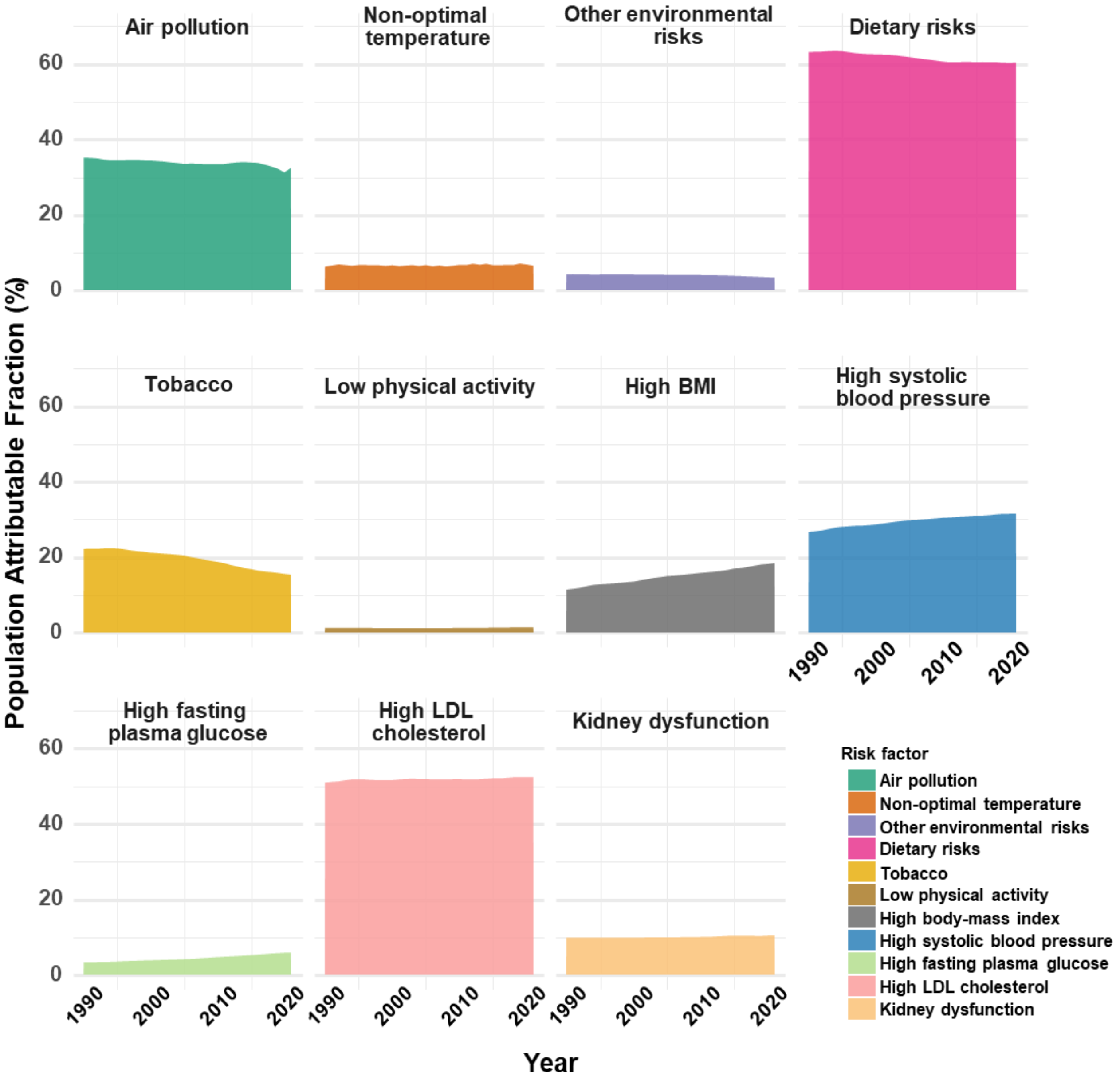


**Supplementary Figure S4.** Temporal trends in population attributable fractions (PAFs) for individual risk factors from 1990 to 2021.

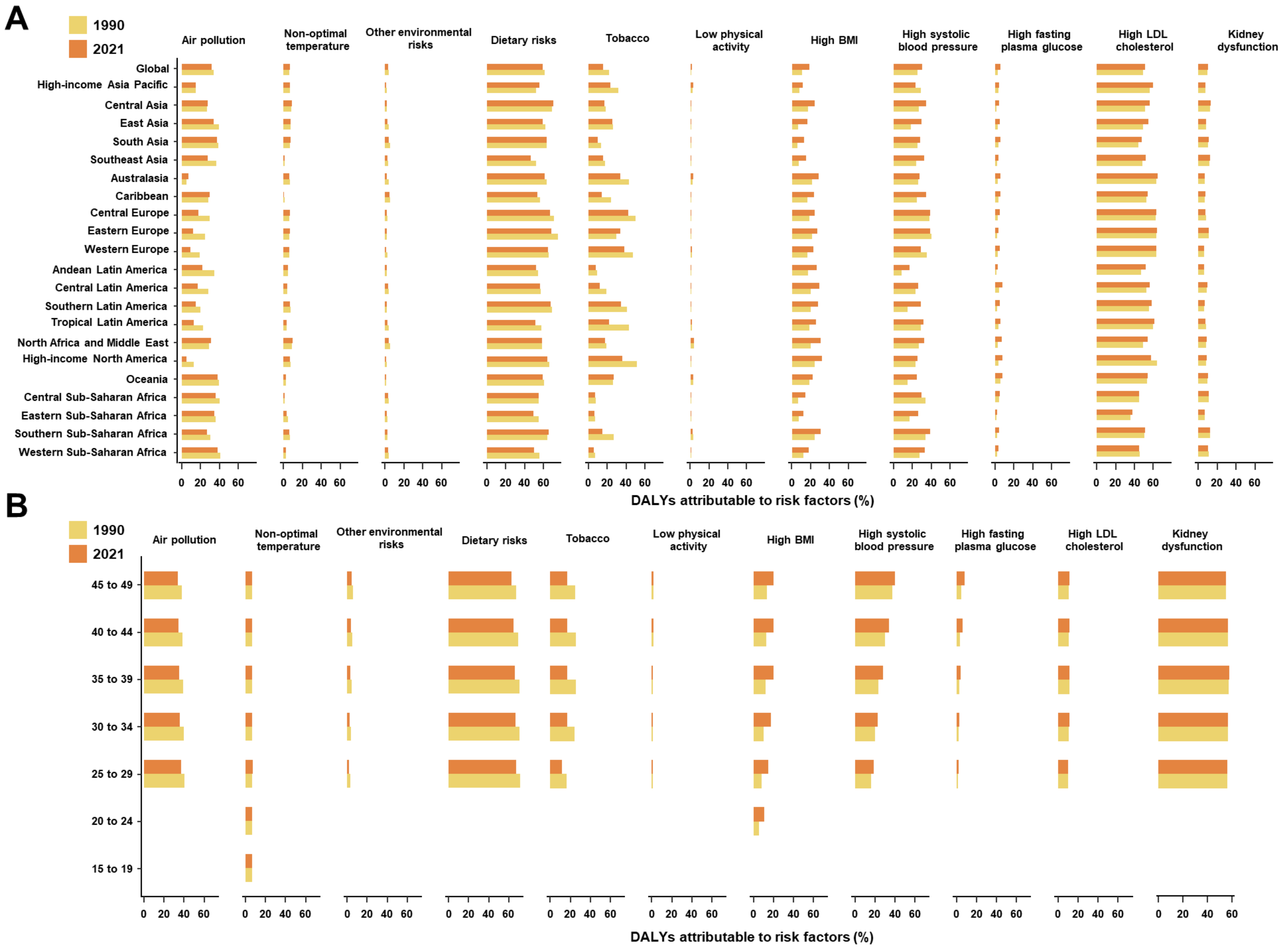


**Supplementary Figure S5.** The proportion of DALYs attributable to risk factors for ischemic heart disease among women of childbearing age by locations (A) and age group globally (B) in 2021. In the GBD 2021, certain risk factors were modeled with lower age restrictions of 20 or 30 years, which resulted in the absence of estimates for these risk factors in age groups such as 15-19 years, 20-24 years, and 25-29 years. DALY=Disability-adjusted life-years.
